# The role of volatiles in plant communication

**DOI:** 10.1111/tpj.14496

**Published:** 2019-09-19

**Authors:** Harro Bouwmeester, Robert C. Schuurink, Petra M. Bleeker, Florian Schiestl

**Affiliations:** ^1^ University of Amsterdam Swammerdam Institute for Life Sciences Green Life Science research cluster Science Park 904 1098 XH Amsterdam The Netherlands; ^2^ Department of Systematic and Evolutionary Botany University of Zürich Zollikerstrasse 107 CH‐8008 Zürich Switzerland

**Keywords:** terpenoids, phenylpropanoids, volatiles, plant−insect/microbe/plant interactions, pollination, biosynthesis, regulation, agriculture

## Abstract

Volatiles mediate the interaction of plants with pollinators, herbivores and their natural enemies, other plants and micro‐organisms. With increasing knowledge about these interactions the underlying mechanisms turn out to be increasingly complex. The mechanisms of biosynthesis and perception of volatiles are slowly being uncovered. The increasing scientific knowledge can be used to design and apply volatile‐based agricultural strategies.

## Significance Statement

Volatiles mediate the interaction of plants with pollinators, micro‐organisms, other plants and herbivores and their natural enemies. The increasing knowledge about the role of volatiles in these interactions, the underlying mechanisms, the biosynthesis and perception of volatiles are reviewed and the perspective for volatile‐based agricultural control strategies discussed.

## Introduction/Background

**Figure 1:**
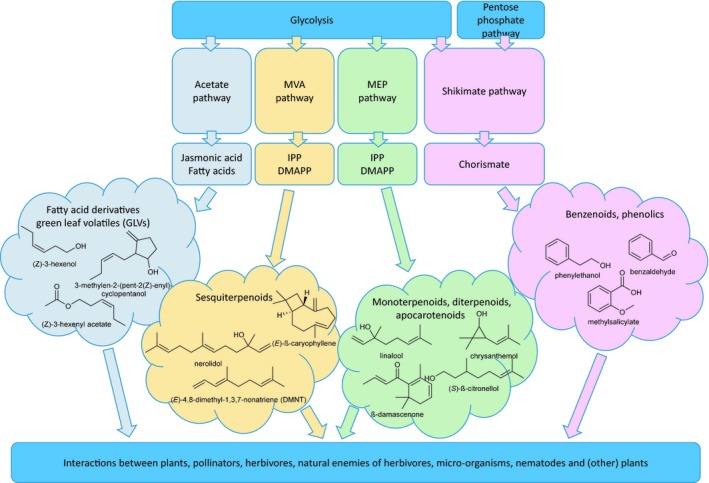
Biosynthetic pathways of the major volatile compound classes and selected examples of volatiles from these classes that are discussed in this review. Fatty acid‐derived VOCs (including GLVs and methyljasmonate) are produced from C18 fatty acids, produced through the acetate pathway that gets its substrate, acetyl‐coA, from glycolysis (Fu *et al*., 2017). Acetyl‐coA from glycolysis is also the substrate for the cytosolic mevalonic acid (MVA) pathway, while pyruvate from glycolysis is the substrate for the plastidic methylerythritol phosphate (MEP) pathway (Beyraghdar Kashkooli *et al*., 2018; Pichersky and Raguso, 2018). The benzenoids and phenylpropanoids are produced through the shikimate pathway that gets its substrates from glycolysis and the pentose phosphate pathway (Widhalm *et al*., 2015; Santos‐Sánchez *et al*., 2019).

Through volatile organic compounds (VOCs), plants are in constant dialogue with the organisms in their environment. This communication is of great importance as it allows plants and the organisms they interact with to tune their growth, development, defence, propagation and life cycle to achieve maximal fitness. Plants themselves but also the organisms in their environment produce VOCs, which belong to various chemical classes, such as the terpenoids, the benzenoids and phenylpropanoids, fatty acid‐derived molecules including the green leaf volatiles (GLVs) and minor classes such as nitriles, (ald)oximes and sulfides (Figure 1). Here we review the recent literature on volatile communication between plants and other organisms as well as the biosynthesis, transport, perception in the receiver organisms, and potential importance for agriculture of these VOCs.

## Analysis and Information Content of VOCs

Volatile organic compounds mediate the interaction between plants and mutualists, pests and pathogenic antagonists and, although not addressed much yet and experimentally challenging, also multi‐trophic interactions. Semiochemicals that function in these interactions can be produced constitutively or in response to outside interactions and stimuli, and this occurs above as well as below ground (Massalha *et al*., 2017). Although there are indications that above‐ and below‐ground semiochemical responses are integrated (Danner *et al*., 2015; Van Dam *et al*., 2018), details on what drives this remain to be elucidated. Although semiochemicals can travel long distances, plant−plant and plant−microbe communication usually take place at relatively short distances, while plant volatiles with a role in plant−insect interaction are perceived at distances of several hundred metres. The resulting extreme dilution, and the large variation in chemical structures and properties of the VOCs pose a challenge to the analysis of the volatiles and their precursors (Fu *et al*., 2017). Another major challenge is to interpret VOC information content, especially across larger spatial scales in which the volatile blend carrying the information becomes increasingly complex through environmental effects (including noise) and community dynamics in an ecosystem (Aartsma *et al*., 2017; Kessler and Kalske, 2018). Comparison of volatile profiles is complicated due to their complexity, but also because the presence/absence and quantity of compounds are not independent measures in case of a shared biosynthetic origin. An interesting approach to deal with this is a so‐called biosynthetically informed pairwise distance measure, calculating compound similarity based on the proportion of shared biosynthetic enzymes (Junker, 2018). In the coming years we will see a strong progress in the interpretation of the effect of volatile blends on biological processes. This development will be fuelled by the development of new high‐throughput behavioural bioassays in combination with high‐throughput VOC analyses and increasing sensitivity of GC‐MS. Upcoming tools such as network analysis, large‐scale data integration, and machine learning are going to provide a breakthrough in the interpretation of the biological meaning of the complex VOC blends (Cuperlovic‐Culf, 2018).

## The Role of VOCs in Pollination

One of the major functions of floral volatiles is the attraction of pollinators, either through triggering innate behavioural responses or by providing a stimulus that can be associated with the presence of rewards (Schiestl and Johnson, 2013; Haverkamp *et al*., 2016a). Pollinator attraction is often surprisingly specific, which can be beneficial as specificity increases the efficiency of pollen transfer between conspecific stigmas. Specificity in pollination can be achieved with floral filters (Raguso, 2008), for example through specialized morphology that prevents access to rewards for most animals, or signals that are only detectable or of interest to few potential pollen vectors. Among signals, colour is rarely highly specific, because most animals have limited abilities to discriminate subtle colour nuances. Therefore, floral scent is typically more important in encoding highly specific signals, because of its chemical complexity, and the high number of different olfactory neurons usually used by insects to detect it (Haverkamp *et al*., 2018). The chemical complexity in floral scent entails interactions between neurons detecting individual chemical components, together producing the sensation of a bouquet, which is different from the sum of the individual components (Strutz *et al*., 2014; Schiestl, 2015). An example for this is scent compounds that modify the attractive function of other molecules, as was shown, for example, in scent bouquets of orchids attracting fragrance‐gathering male euglossine bees (Milet‐Pinheiro *et al*., 2018). Floral scent is mostly detected by insects with neurons located on the antennae, but recently olfactory neurons on the tip of the proboscis have also been characterized (Haverkamp *et al*., 2016b).

Some of the most specific chemical signals are those that mimic non‐floral items of interest to potential pollinators, such as oviposition substrates or mating partners (Johnson and Schiestl, 2016). Such signals are usually not attractive or not even detectable to ‘normal’ floral visitors that visit flowers for nectar and/or pollen, and therefore filter out a large number of potential visitors. Even more so, scent bouquets emitted by mimetic flowers are sometimes more specific than the volatiles emitted by the model they mimic. One curious example comes from the flowers of *Rafflesia cantleyi* (Wee *et al*., 2018) that emit a smell mimicking rotten meat and attracting flies that normally oviposit in such substrates, as pollinators. In the field, where this *Rafflesia* species flowers, rotting meat was found to attract nine species of flies, whereas only five species were found on *Rafflesia* flowers, and of those, only a single species, the blowfly *Chrysomy chani*, comprised 97% of all flower visitors and was the only species carrying pollen. On traps with rotting meat, *C. chani* represented only 25% of all flies caught, suggesting that floral volatiles of *Rafflesia cantleyi* effectively filter the available carrion‐fly pollinator community (Van der Niet *et al*., 2011). The two main compounds of *Rafflesia*'s floral bouquet are dimethyl disulfide (DMDS) and dimethyl trisulfide (DMTS), products of bacterial breakdown of methionine and cysteine in meat, that are typically found in the floral emission of floral carrion mimics (Martin *et al*., 2017; Du Plessis *et al*., 2018; Wee *et al*., 2018). Many species of blowflies are attracted to these volatiles, but *Rafflesia* flowers also produce terpenoids, benzenoids and fatty acid derivatives, which may have a modulating function leading to higher specificity of the whole bouquet.

A similar case has recently been recorded in *Ceropegia mixta*, which is mostly pollinated by the house fly *Musca domestica* (Du Plessis *et al*., 2018). *M. domestica* is usually not strongly associated with carrion but also oviposits in dung, suggesting that *C. mixta* uses mixed carrion/faeces mimicry (Jürgens *et al*., 2013). The floral scent of this plant is also dominated by DMDS and DMTS, in combination with a complex blend of other compounds. A fascinating twist is the fact that even though vision models suggest that for flies the colour of the flowers is indistinguishable from the background, bioassays suggest that there is a visual component of attraction. Whereas odour alone was sufficient to attract flies, combined olfactory and visual signals attracted more flies. This finding is paralleled by data from the *Rafflesia* system, in which floral scent alone was insufficient to trigger landing behaviour of the pollinators in wind tunnel bioassays, suggesting visual signals are needed to guide insects to carrion flowers (Du Plessis *et al*., 2018).

A well known, and highly specific example of floral mimicry is sexual mimicry, in which flowers imitate mating signals and are pollinated by sexually aroused male insects during a mating attempt (Johnson and Schiestl, 2016). This type of pollination system is most commonly found among Australian orchids, in which a minimum of 11 genera are (at least partly) pollinated in this way. In some of these groups, exciting progress has been made in recent years in terms of identifying the pollinator‐attracting floral scent compounds (i.e. the mimicry of the sex pheromone), and several unusual compounds have been identified. For example, (methylthio)phenols, up to now only known as natural products produced by bacteria, were identified as pollinator‐attracting signals in the orchid genus *Caladenia* (Bohman *et al*., 2018b). *Caladenia crebra* produces four types of (methylthio)phenols, and a 10:1 blend of two of these compounds attracts and releases copulation attempts in its legitimate pollinator, a thynnine wasp. Interestingly, a 1:1 blend of the same compounds attracts another, as yet undescribed, thynnine wasp (*Campylothynnus* sp. A), which is the pollinator of *Caladenia attingens* subsp *attingens*, demonstrating the importance of specific ratios of active volatiles for the specificity of pollinator attraction in sexual mimicry.

**Figure 2:**
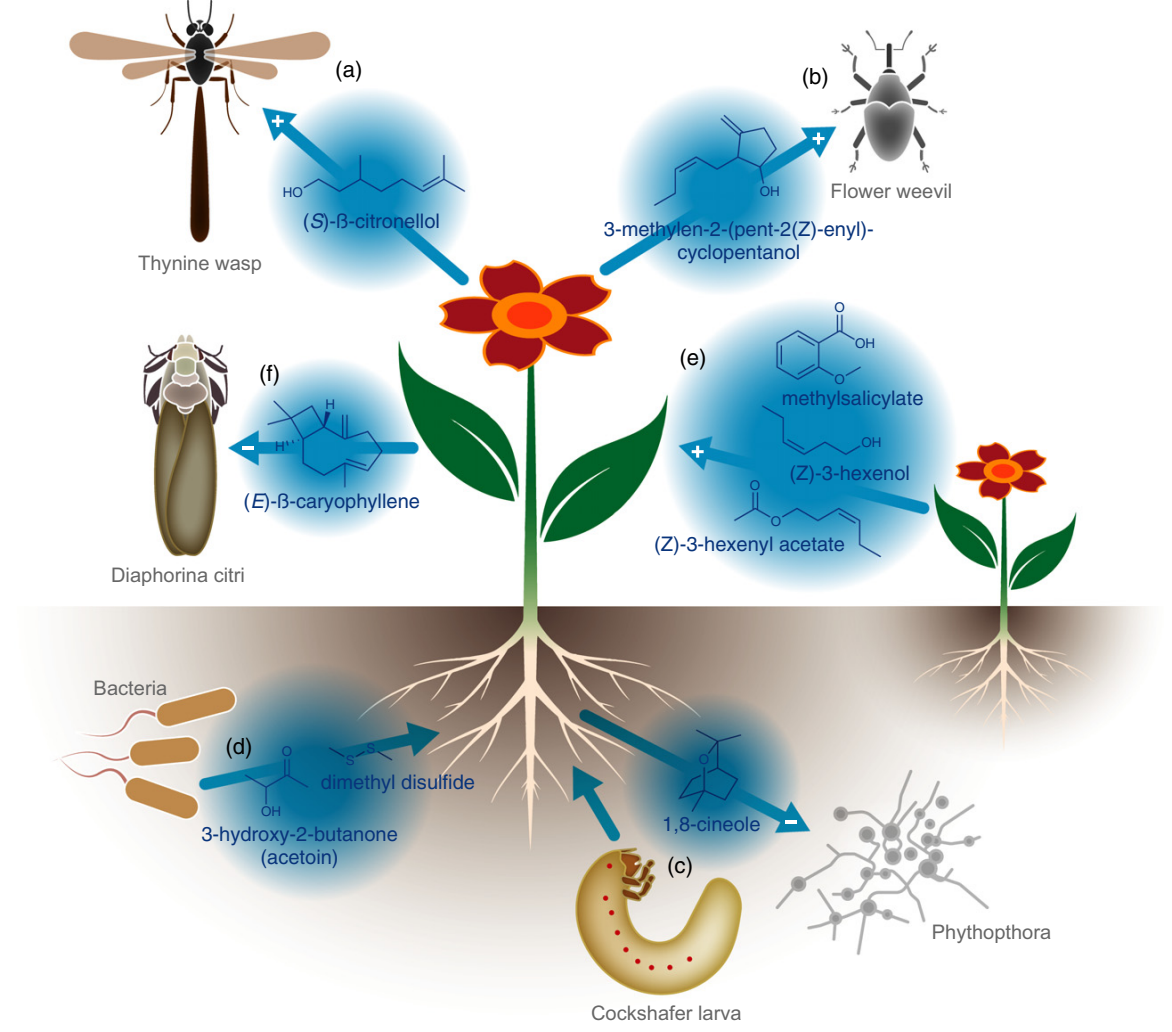
Communication between plants and other organisms. (a) Flowers of *Caladenia plicata* emit (*S*)‐β‐citronellol as attractant of pollinator thynnine wasps (Bohman *et al*., 2018a). (b) *Carludovicoideae* plant species emit the unusual 3‐methylen‐2‐(pent‐2(*Z*)‐enyl)‐cyclopentanol to attract flower weevils (Teichert *et al*., 2018). (c) Cockchafer larvae feeding on roots of *Populus* spp. induce the emission of monoterpenes such as 1,8‐cineole, which has an inhibitory effect on *Phytophtora cactorum* (Lackus *et al*., 2018). (d) Rhizobacteria can stimulate root growth and induce resistance through the production of volatiles such as 3‐hydroxy‐2‐butanone (acetoin) and dimethyl disulfide, respectively (Fincheira and Quiroz, 2018). (e) Upon infection and/or herbivory, plants emit for example (*Z*)‐3‐hexenol, (*Z*)‐3‐hexenyl acetate and methyl salicylate. Receiver plants show remarkably specific responses to these volatiles (Park *et al*., 2007; Sugimoto *et al*., 2014; Cofer *et al*., 2018b; Erb, 2018; Hu *et al*., 2018b). (f) (*E*)‐β‐caryophyllene repels HLB transmitting *Diaphorina citri*. Naturally (*E*)‐β‐caryophyllene emitting plant species such as guave, the use of (*E*)‐β‐caryophyllene dispensers and metabolic engineering of (*E*)‐β‐caryophyllene production can potentially be used to protect citrus trees against HLB (Mafra‐Neto *et al*., 2013; Alquézar *et al*., 2017).

In another paper, Bohman and colleagues show a different mechanism of specificity, in which pollinators of sexual mimics strongly discriminate between structural isomers of active volatiles (Bohman *et al*., 2018a). This was shown to be the case for *Caladenia plicata*, which employs two unusual and biosynthetically unrelated volatiles as pollinator attractants, (*S*)‐β‐citronellol and 2‐hydroxy‐6‐methylacetophenone. When (*S*)‐β‐citronellol was replaced by its enantiomer (*R*)‐β‐citronellol or 2‐hydroxy‐6‐methylacetophenone by one of its regio‐isomers in bioassays with synthetic volatiles, the volatile blend lost almost all of its attractiveness (Figure 2a). In a generalist pollinator searching for food on flowers, namely honey bees, a different result was found, as worker bees only poorly discriminated between isomers of different volatiles, even when they were paired with aversive stimuli like bitter taste (Aguiar *et al*., 2018). A fascinating aspect of specific floral signals is the potential of generalist predators to eavesdrop on such signals to optimize prey catching. On a Balearic island, for example, lizards use carcasses, but also the carrion‐mimicking flowers of *Helicodorus muscivorus*, as a lurking site to catch flies (Perez‐Cembranos *et al*., 2018). A very similar example, but without involving mimetic flowers, is represented by crab spiders that wait for their prey at flowers (Knauer *et al*., 2018). Both lizards and crab spiders are attracted by floral scent, which makes sense because a ‘sit and wait’ predator is likely to be more successful when using the same signals as its prey to identify potential hunting sites. Both lizards and crab spiders eat or repel pollinators and therefore have a negative effect on the pollination success of the plant. In both cases, however, there is also a positive effect on plant fitness, as lizards act as fruit dispersers of *Helicodorus* and crab spiders also feed on florivores. This potential fitness increase counterbalances selection on plants to avoid eavesdropping predators and are likely to stabilize these associations. Another, often highly specific and scent‐driven, pollination system is nursery pollination, in which pollinators oviposit into flowers and the pollinators’ larvae consume floral tissue of developing seeds. Jackfruit (*Artocarpus heterophyllus*) is a monoecious crop pollinated by gall midges that visit male and female inflorescences, but oviposit only into male, fungus‐infected inflorescences (Gardner *et al*., 2018). Male inflorescences are typically infested by a fungus after blooming. Female inflorescences are deceptive to pollinators as they do not provide reward, even though they produce more volatiles than the male flowers. Floral scent of both male and female inflorescences is dominated by three methyl esters that release responses in the midges’ olfactory neurons and also occur in the fruits. Highly specific, unusual scent compounds – such as (*E*,*E*)‐α‐farnesene‐2(3),9(10)‐diepoxyde and 3‐methylen‐2‐(pent‐2(*Z*)‐enyl)‐cyclopentanol – were found in three *Carludovicoideae* plant species with nursery pollination (Teichert *et al*., 2018) (Figure 2b). The floral staminoids of these plant species produce scent and elevated temperature to attract flower weevils that oviposit into the staminoids. In summary, highly specific associations between flowers and insects are typically mediated by chemical signals, that act as floral filters together with visual signals, floral morphology and specific types of reward.

## The Role of VOCs in the Interaction Of Plants With Herbivorous Insects

The unique constitutive volatile blend produced by plants is used by mutualists such as pollinators, but also by antagonist such as herbivorous insects who use volatiles to identify and home‐in on a host. The host blend consists of major and minor constituents, but the most abundant compounds do not automatically determine the response to the blend as a whole (McCormick *et al*., 2012). The olfactory system of insect antennae can discriminate patterns and use this to determine biological relevance, that is host suitability or unsuitability. This odour‐mediated behaviour appears to be innate in insects (Hatano *et al*., 2015). Within a plant community, the chemical diversity in constitutive volatiles between individuals can be large enough to drive herbivore preference‐behaviour (Clancy *et al*., 2018).

Herbivore‐induced plant volatiles (HIPVs) are blends of volatiles – mostly GLVs, terpenes and aromatic compounds – that a plant produces in response to herbivory (McCormick *et al*., 2012). HIPVs are released after tissue disruption; however, albeit at low levels, some are also emitted constitutively into the plants headspace (Holopainen and Gershenzon, 2010). HIPVs serve as signals to attract natural enemies of herbivores such as parasitoids and predators (Aartsma *et al*., 2017; Gasmi *et al*., 2018; Turlings and Erb, 2018). Cockchafer larvae feeding on roots of *Populus* spp. induced the emission of a number of monoterpenes including 1,8‐cineole, which had an inhibitory effect on *Phytophtora cactorum* (Lackus *et al*., 2018) (Figure 2c). Turlings and Erb (2018) question the common notion that HIPVs are signals produced to attract the pest enemy or as ‘cry for help’, as there is no evidence for directed evolution or a fitness benefit (Turlings and Erb, 2018). The signal itself that is produced in direct response to the attacker is associated with prey so accurately that parasitoids and predators have evolved to rely on the volatile cues to such an extent that they, for example, have even lost vision.

## The Role of VOCs in the Interaction of Plants With Micro‐Organisms

Not only plants produce volatiles in response to interspecies interaction, but also micro‐organisms associated with plants are a source of volatile (and non‐volatile) compounds that can alter plant physiological and metabolic responses (Piechulla *et al*., 2017; Wenke *et al*., 2019). *Bacillus*,* Pseudomonas*,* Arthrobacter*,* Fusarium* and *Alternaria* rhizobacteria, for example, can stimulate root growth through the production of volatiles such as 3‐hydroxy‐2‐butanone (acetoin), 2,3‐butanediol and 2‐pentylfuran (Fincheira and Quiroz, 2018) (Figure 2d). Others can induce resistance and tolerance in plants such as acetoin, dimethyl disulfide, 3‐pentanol and 6‐pentyl‐α‐pyrone (Fincheira and Quiroz, 2018) (Figure 2d). This shows that plants do not only produce volatiles, but also perceive them, and respond to them with altered growth or defence signalling (Schenkel *et al*., 2018). This is further discussed below under ‘Molecular mechanisms underlying VOC perception’. Microbe‐induced plant volatiles can inhibit microbial growth and act as local bacteriostatic agents (Sharifi *et al*., 2018) as discussed for 1,8‐cineole and *P. cactorum* above (Lackus *et al*., 2018). Indoles and green leaf volatiles (GLVs) can affect membrane integrity of bacteria on the plant surface and interfere with bacterial quorum sensing, thereby deregulating bacterial virulence (Joshi *et al*., 2016). In response to pathogen attack, plants do not only activate their own defence response, but the infection can also induce volatile emission into the headspace that can lead to an immune response in neighbouring plants (Riedlmeier *et al*., 2017) (Figure 2e). However, when multiple volatile signals have to be integrated, the immune (or defence) response can also be reduced. Hormonal cross‐talk has a role in balancing trade‐offs, which can also result in the suppression of volatile emission (Martínez‐Medina *et al*., 2017; Erb, 2018). Therefore, plants can produce specific volatiles upon microbial interactions but, in turn, microbes have been shown to be able to suppress VOC biosynthesis through the use of effectors (Sharifi *et al*., 2018). Microbes, in combination with their host plant, can also mediate tritrophic interactions (Shikano *et al*., 2017). For example, viruses that depend on a (mobile) vector, may alter plant volatile biosynthesis in order to recruit the vector insect. The 2b protein of cucumber mosaic virus manipulates aphid behaviour by modifying the host volatile profile (Tungadi *et al*., 2017; Wu *et al*., 2017). Even further complicating the story, herbivorous insects carry facultative endosymbionts that can attenuate the production of predator‐attracting volatiles emitted by the plant under attack (Frago *et al*., 2017). The role of micro‐organisms in the volatile communication of plants also extends to flower volatiles. Microbes can colonize the nectar and change the volatile blend of a flower, which may alter floral visitation and hence plant reproduction (Vannette and Fukami, 2016; Rering *et al*., 2018). In conclusion, the dialogue between plants and microbes is complex and deciphering the infochemical exchange between plants and beneficial as well as pathogenic microbes remains of unaltered interest for application in (crop)plant production, as further discussed below.

## The Role of VOCs in Plant−Plant Interaction

An intriguing function of volatiles is their signaling role in and between plants, a phenomenon first described already more than 35 years ago (Baldwin and Schultz, 1983; Rhoades, 1983). When attacked by herbivorous arthropods, plants often emit a different blend of volatiles, both qualitatively and quantitatively and this blend of volatiles can initiate responses in systemic leaves and neighbouring plants. As such, plants are also indicated as having a certain dialect or chemotype (Karban *et al*., 2014a, 2016). Many studies have shown that this volatile blend can activate or prime defence responses, for reviews see for example Heil and Karban (2010) and Ameye *et al*. (2018). A recent meta‐analysis showed that adjacent plants exposed to volatiles from damaged neighbours become more resistant to herbivores (Karban *et al*., 2014b). However, tomato plants infested by whiteflies (*Bemisia tabaci*) produce a volatile blend that makes neighbouring plants more susceptible to whiteflies (Zhang *et al*., 2019). Whether this is an adaptive response, as it might make the plants more resistant to the viruses transmitted by the whiteflies, remains to been seen, but it indicates that not all HIPV blends result in more resistance to herbivores in the neighbours.

## Molecular Mechanisms Underlying VOC Perception

An intriguing function of volatiles is their signaling role in and between plants, and particularly how plants can perceive these signals (Cofer *et al*., 2018b; Erb, 2018). For examples of this, authors normally refer to the gaseous hormone ethylene and its receptor (Schaller and Bleecker, 1995) and suggest that other volatiles may also be detected by receptors in plants. However, receptors for other volatiles have remained elusive so far. Still, plants show an often surprisingly specific response to different volatiles. Therefore perhaps one has to look at alternative scenarios of which one example is the perception of CO_2_ by plants in guard cells. These cells in Arabidopsis have an S‐type anion channel, SLOW ANION CHANNEL1 (SLAC1) that has bicarbonate‐interaction sites (Yamamoto *et al*., 2016; Zhang *et al*., 2018a). It is conceivable that other ion channels have interaction sites for particular volatiles as well that can change their activity just as CO_2_/HCO_3_^−^ can change SLAC1 activity and stomatal movement. Indeed, the first response to volatiles that can be measured in plants is a change in plasma membrane potential and cytosolic calcium flux (Zebelo *et al*., 2012). Very recently, the transcriptional co‐repressor TOPLESS‐like (TPL) protein was identified to bind volatile sesquiterpenes, that is caryophyllene analogues (Nagashima *et al*., 2018). TPL proteins are known to regulate various hormonal responses such as those induced by jasmonic acid (Pauwels *et al*., 2010). This suggests that nuclear proteins can be involved in volatile sensing, and opens up an interesting field of research while at the same time raising the question how these volatiles are transported to establish a relatively high concentration in the nucleus.

Also enzymes have been demonstrated to play a role in transport and perception of volatiles within or between plants. The most well known intracellular signalling volatile is methylsalicylate (MeSA) that is transported via the phloem from a tobacco (*Nicotiana tabadefenccum*) leaf infected with tobacco mosaic virus (TMV) to a non‐infected, systemic leaf to induce resistance (Park *et al*., 2007). Accumulation of high levels of salicylic acid (SA) in TMV‐infected leaves results in the production of MeSA and the inhibition of a MeSA esterase in the local leaf (Figure 2e). In the systemic leaf, SA levels are low and the transported MeSA is converted to SA by the esterase resulting in SA production and resistance. In plant−plant communication it has recently become clear that in receiver tomato plants, the GLV (*Z*)‐3‐hexenol can be glycosylated to (*Z*)‐3‐hexenylvicianoside, which induces defence responses (Sugimoto *et al*., 2014) (Figure 2e). Intriguingly this particular glycosylation seems very plant species specific, as it does not occur in Arabidopsis, even though the latter also glycosylates many different volatile alcohols – GLVs, phenylpropanoids, terpenes – upon exposure (Sugimoto *et al*., 2015).

Plants show remarkably specific responses to different volatiles (Cofer *et al*., 2018b; Erb, 2018). Recent findings show that dual‐volatile exposed plants respond differently than plants exposed to single volatiles. Maize (*Zea mays*) plants respond with stronger defences to exposure with both indole and the GLV (*Z*)‐3‐hexenyl acetate than to the single volatiles (Hu *et al*., 2018b) (Figure 2e). This adds another layer of complexity and might explain some of the very specific responses that have been observed. For instance, plant−plant communication in the shrub *Baccharis salicifolia* is specific for a particular aphid species: induced resistance in neighbours is only obtained against the same aphid (Moreira *et al*., 2018). This specificity can be assigned to the different volatile blends induced by the different aphids. Similarly, as in maize, a blend of synthetic volatiles mimicked this response better than the individual compounds. This seems to indicate that plants have multiple mechanisms to detect volatiles as already suggested by Cofer *et al*. (2018b).

Many new aspects of responses to volatiles and volatile signal transduction in plants are being discovered. GLVs, for example, can protect maize seedlings against cold stress (Cofer *et al*., 2018a) and particular GLVs can induce stomatal closure thereby enhancing resistance against *Pseudomonas syringae* pv. *tomato* infection (López‐Gresa *et al*., 2018). This GLV‐induced priming comes at a cost for the plants as it reduced growth rates (Engelberth and Engelberth, 2019). Other interesting findings are that touch‐induced volatiles can synchronize defences in neighbouring plants (Markovic *et al*., 2018) and that goldenrod (*Solidago altissima*) responds to (*E*,*S*)‐conophthorin, a volatile produced by a specialist herbivore, the goldenrod gall fly (*Eurosta solidaginis*) (Helms *et al*., 2017). This brings another dimension to the field of plant−herbivore interactions. Regarding signalling, an advanced publication (Ye *et al*., 2018) indicates a role for mitogen‐activated kinases and WRKY transcription factors in indole‐mediated priming, similar to what has been shown for GLV signaling in Arabidopsis (Ameye *et al*., 2018), perhaps pointing to signalling commonalities. Finally, one very intriguing aspect is the response of plants to microbial volatiles (Ryu *et al*., 2003; Sharifi and Ryu, 2018). With, for instance, clear growth responses to specific volatiles as read out, genetic screens to identify potential receptors and signal transduction cascades should be more feasible than with volatiles in plant−plant communication (Scala *et al*., 2017).

## VOC Biosynthesis

Intriguingly, the VOCs that mediate the communication of plants and other organisms belong to a range of different compound classes such as fatty acid‐derived molecules (including GLVs), terpenoids, benzenoids, and phenylpropanoids, and minor classes such as nitriles, (ald)oximes and sulfides. Fatty acid‐derived VOCs (including GLVs) are produced from the C18 unsaturated fatty acids, linoleic and linolenic acid. These are produced through the acetate pathway that gets its substrate, acetyl‐coA, from glycolysis (Fu *et al*., 2017) (Figure 1). Through a series of oxidation and reduction steps and acetylation these fatty acids are converted to aldehydes, alcohols and acetates, such as (*Z*)‐3‐hexenol and (*Z*)‐3‐hexenyl acetate (Figure 1). Methyl jasmonate biosynthetically originates from linolenic acid through multiple enzymatic steps (Li *et al*., 2005). Methyl jasmonate is formed through methylation of jasmonic acid by jasmonic acid carboxyl methyltransferase (Cheong and Choi, 2003).

Acetyl‐CoA from glycolysis is also the substrate for the cytosolic mevalonic acid (MVA) pathway, while pyruvate from glycolysis is the substrate for the plastidic methylerythritol phosphate (MEP) pathway (Beyraghdar Kashkooli *et al*., 2018; Pichersky and Raguso, 2018). These two pathways both produce the isoprenoid precursors, isopentenyl diphosphate (IDP) and its isomer, methylallyl diphosphate (DMADP). IDP and DMADP are condensed by prenyl transferases, such as geranyl diphosphate (GDP) synthase, farnesyl diphosphate (FDP) synthase, and geranylgeranyl diphosphate (GGDP) synthase to produce GDP (C10), FDP (C15), and GGDP (C20), respectively. FDP from the MVA pathway is the precursor for cytosolic sesquiterpenoid biosynthesis catalyzed mainly by sesquiterpene synthases, cytochrome P450s, alcohol dehydrogenases and reductases. GDP from the MEP pathway is the precursor for plastidic monoterpenoid and carotenoid formation. Monoterpenoid formation is mostly catalyzed by monoterpene synthases, cytochrome P450s, alcohol dehydrogenases and reductases (Beyraghdar Kashkooli *et al*., 2018; Pichersky and Raguso, 2018). GGDP is the precursor for carotenoid formation through a series of enzymatic steps, involving a.o. phytoene synthase and phytoene desaturase. Carotenoids such as β‐carotene are precursors for the production of volatile apocarotenoids through oxidative cleavage, catalyzed by carotenoid cleavage dioxygenases (Liang *et al*., 2018) (Figure 1).

The benzenoids and phenylpropanoids are derived from the shikimate pathway, which starts with the condensation of phosphoenolpyruvic acid from glycolysis, and d‐erythrose‐4‐phosphate, from the pentose phosphate cycle (Widhalm *et al*., 2015; Santos‐Sánchez *et al*., 2019) (Figure 1). Then six more enzymatic steps result in the formation of the key branch‐point compound, chorismic acid, the final product of the shikimate pathway and the common precursor for all (volatile) phenolics. The volatile phenolics are represented by benzenoids (C6–C1), such as benzaldehyde, methyl salicylate and benzyl benzoate, which are formed from cinnamic acid; C6−C2 compounds such as 2‐phenylethanol and phenyl acetaldehyde which are produced from phenylalanine; and C6−C3 compounds such as eugenol, methyl eugenol, and chavicol which are produced from 4‐coumaroyl‐CoA (Widhalm *et al*., 2015) (Figure 1).

The (ald)oximes are amino acid‐derived metabolites and include volatiles, such as 2‐ and 3‐methylbutyraldoxime. The volatiles are herbivory‐induced or flower volatile blend constituents. See (Sørensen *et al*., 2018) for a recent review. In addition to these canonical VOC biosynthetic pathways occasionally unexpected biosynthetic solutions appear in the literature, showing how immensely intricate volatile biosynthesis has evolved (Sun *et al*., 2016). The enzyme responsible for geraniol biosynthesis in rose, for example, turned out not to be a monoterpene synthase but a Nudix hydrolase (a diphosphohydrolase) (Magnard *et al*., 2015). More function‐based overviews of volatile biosynthesis are given in papers on the role of volatiles in plant−herbivore interaction including the perception by plants of herbivory and downstream signalling (Stahl *et al*., 2018), the role of volatiles in plant−microbe interaction (Sharifi *et al*., 2018) and on flower volatile biosynthesis (Ramya *et al*., 2017; Wong *et al*., 2017). Ryu and co‐workers review the induction of volatiles by micro‐organisms and the effects that these volatiles have on plants, showing that microbes are true manipulators of their plant host (Sharifi *et al*., 2018).

With regard to progress in our understanding of the biosynthesis of herbivory‐induced volatiles, Liu *et al*. (2018a) identified two cytochrome P450s, GhCYP82L1 and GhCYP82L2, that can both catalyze the formation of the C‐11‐homoterpene (*E*)‐4,8‐dimethyl‐1,3,7‐nonatriene (DMNT) and the C‐16‐homoterpene (*E*,*E*)‐4,8,12‐trimethyltrideca‐1,3,7,11‐tetraene (TMTT) from nerolidol and geranyllinalool, respectively (Liu *et al*., 2018a). These cytochrome P450s are highly homologous to AtCYP82G1 that is responsible for TMTT production from geranyllinalool in Arabidopsis (Lee *et al*., 2010). The absence of specificity for either nerolidol or geranyllinalool in the cotton P450s contrasts with maize in which two P450s, ZmCYP92C5 and ZmCYP92C6, are specific for either DMNT or TMTT production, respectively (Richter *et al*., 2016).

With regard to progress in our understanding of the biosynthesis of flower volatiles, Wong *et al*. describe a study into the biosynthetic origin of the putatively fatty acid‐derived, UV‐B induced, chiloglottones, 2,5‐dialkylcyclohexan‐1,3‐diones, that are employed by Australian sexually deceptive orchids of the genus *Chiloglottis* to attract male wasp pollinators (Wong *et al*., 2018). Using transcriptomics data, they identified upregulation of fatty acid biosynthesis and β‐oxidation in flowers and flower parts producing chiloglottones. Using an inhibitor of fatty acid biosynthesis, they demonstrated that this pathway is indeed involved in the production of the precursor of these chiloglottones.

Zhou and colleages used stable isotope‐labelled phenylalanine and acetophenone, and the isolation and characterization of a short chain dehydrogenase, to show that the acetophenone is the preferred substrate for phenylethanol production in tea flowers (Zhou *et al*., 2018). This provides a fourth biosynthetic route to phenylethanol in plants (Sun *et al*., 2016). In a follow‐up study they show that two different SDRs are responsible for the production of the two stereoisomers of phenylethanol that are present in the emitted volatiles of tea flowers (Zhou *et al*., 2019) (Figure 1). In rose, a eugenol synthase was identified and characterized, and shown to be highly similar to other eugenol synthases from for example *Clarkia brewerii*,* Ocimum basilicum* and *Petunia hybrida* (Yan *et al*., 2018). Although the core β‐oxidative pathway leading to benzoic acid has been elucidated (Qualley *et al*., 2012), Adebesin and colleagues recently added another player, a peroxisomal thioesterase, PhTE1, that plays an auxiliary role in *Petunia hybrida* flowers (Adebesin *et al*., 2018). With its capability to hydrolyze aromatic acyl‐CoA esters, the authors concluded that PhTE1 exerts control on the flux between β‐oxidation in the peroxisomes and phenylpropanoid biosynthesis in the cytosol.

For the identification of genes involved in volatile biosynthesis several different strategies are being used. An example of a genomics approach is the work by Kumar *et al*. (2018) who mined the genome sequence of *Ocimum sanctum* for terpene synthases (Kumar *et al*., 2018). They identified 47 putative terpene synthases. To get a better insight in their involvement in the biosynthesis of the *O. sanctum* terpenoids additional analyses will be necessary, such as transcriptomics preferably in combination with chemical analysis (and followed by functional characterization through heterologous expression). Transcriptome analysis through RNA sequencing is increasingly being used to identify genes involved in volatile biosynthesis. Fan *et al*. (2018), Huang *et al*. (2018) and Tian *et al*. (2018) used RNA‐seq to try to pinpoint genes that are involved in flower volatile biosynthesis in *Polianthes tuberosa*,* Freesia hybrida* and wintersweet, respectively, that emit benzenoids and terpenoids, mostly terpenoids and terpenoids and benzenoids, respectively (Fan *et al*., 2018; Huang *et al*., 2018; Tian *et al*., 2018). All three base their selection of gene candidates on homology and expression patterns. Although in these studies chemical analysis was also carried out, these data were not used to the maximum to further narrow down and/or support their candidate genes and the studies therefore remain rather descriptive. A more advanced network (co‐expression) analysis with RNA‐seq and GC‐MS results is very powerful in identifying promising candidate genes and will therefore shorten the gene discovery process substantially (He *et al*., 2018). A next step in this process will be the use of metabolomics as exemplified for strawberry (Haugeneder *et al*., 2018), which will allow correlation analysis also with non‐volatile precursors and/or further functionalized non‐volatile downstream products.

With these advanced approaches, the discovery process of biosynthetic genes will further speed up. The elucidation of biosynthetic pathways of VOCs – in combination with the possibility to genetically knock out or knock in the production of specific volatiles through (transient) transformation in combination with behavioural assays – will remain an important tool in elucidating the exact role of individual VOCs in the communication of plants with other organisms.

## Regulation of VOC Biosynthesis

Perhaps even more than for the biosynthetic genes, we are just scratching the surface in which our understanding of the regulation of VOC production is concerned. VOC production in flowers and upon herbivory, for example, is tightly controlled and regulated, developmentally in the case of flower volatiles, biochemically in the case of herbivores. Plants recognize herbivory from damage by herbivore associated molecular patterns (HAMPS), present in the oral secretions of the herbivore, or damaged associated molecular patterns (DAMPS), endogenous plant signals (Duran‐Flores and Heil, 2016). This leads to the production of the defence‐regulating hormones, predominantly jasmonoyl isoleucine (JA‐Ile), and the induction of volatile production, with other hormones also playing a role, while herbivores try to produce effector proteins to counteract this induction (Vos *et al*., 2013; Kant *et al*., 2015; Villarroel *et al*., 2016).

In flowers, volatile production transcription factors of especially the MYB family have been demonstrated to regulate biosynthesis, especially of the benzenoids/phenylpropanoids, as reviewed by Ramya *et al*. (2017). The importance of MYB TFs in regulating flower scent is supported by the discovery of a MYB TF, LhODO1, in lily which seems to regulate volatile benzenoid/phenylpropanoid production (Yoshida *et al*., 2018). LhODO1 showed a diurnal rhythm in tune with the expression of shikimate pathway and PAL genes. LhODO1 expression was high in the strongly scented oriental hybrids and very low in wild lilies. Although the role of the bHLH (basic helix−loop−helix) transcription factors in regulating plant‐specialized metabolism has become quite clear (Goossens *et al*., 2017), recent papers also point to their role in regulating volatile sesquiterpene production in Arabidopsis (Hong *et al*., 2012) and in volatile mono‐ and sesquiterpene biosynthesis in the type VI glandular trichomes of tomato (Xu *et al*., 2018). By using stable transformation in maize and transient activation assays in citrus, a role for AP2/ERF transcription factors in regulating a terpene synthase was illustrated (Li *et al*., 2015, 2017). Similarly, in kiwi NAC transcription factors were shown to bind to the promoter of terpene synthase 1 (Nieuwenhuizen *et al*., 2015). To allow for the identification of TFs that regulate terpene biosynthesis in the traditional Chinese medicinal plant *Amomum villosum*, He *et al*. (2018) used a combination of RNA sequencing and volatile analysis after induction by methyl jasmonate (MeJA) (He *et al*., 2018). Network analysis revealed correlations between putative terpene synthase Unigenes, terpene abundance and candidate WRKY TFs. Further analyses will be needed to prove that these WRKYs indeed regulate terpene biosynthesis.

On top of regulation by classical TFs, specialized metabolite biosynthesis is likely to involve transcriptional and post‐transcriptional silencing, that is regulation via endogenous small non‐coding RNAs (sRNAs). Although a substantial number of sRNAs has been computationally predicted to target genes in specialized metabolic pathways, very few have so far been experimentally validated (Kortbeek *et al*., 2018). microRNAs (miRNAs), although quantitatively by far a minor type amongst the total of small RNAs encountered, have been studied most extensively. In relation to specialized metabolites, there appears to be a pivotal role for miRNA156b (Schwab *et al*., 2005; Gou *et al*., 2011; Yu *et al*., 2015). Yu *et al*. (2015) showed that miR156b targets an SPL9‐type transcription factor that directly binds the promoter of TPS21, thereby affecting (*E*)‐β‐caryophyllene biosynthesis in Arabidopsis (Figure 1). With that finding and the insight in the role of the conserved miR156 in developmental processes, the authors revealed a link between development and volatile biosynthesis, a link that was confirmed by Singh and Sharma (2017) studying the role of miR156b in cucurmin biosynthesis and rhizome development (Singh and Sharma, 2017).

Co‐transcription, another intriguing form of regulation – through alternative splicing – is described by Liu *et al*. (2018b) in tea (Liu *et al*., 2018b). The authors found that *CsLIS/NES* is transcribed into two splicing forms: *CsLIS/NES‐1* and *CsLIS/NES‐2* with the latter lacking a 305 bp‐fragment at the N‐terminus and is why the encoded protein is targeted to the cytosol, while CsLIS/NES‐1 is targeted to the plastids. Upon expression in *E. coli*, both splice forms produce nerolidol and linalool from FDP and GDP, respectively (Figure 1). Expression in tobacco and silencing in tea leaves suggested that CsLIS/NES‐1 *in vivo* acts as linalool synthase and CsLIS/NES‐2 as a nerolidol synthase, although the experimental evidence for the latter is not very strong, possibly due to conjugation of the produced nerolidol (Houshyani *et al*., 2013). Intriguingly, only one of the two splice forms, CsLIS/NES‐1, was induced by MeJA (Liu *et al*., 2018b). It would be interesting to see if this matches with the induction of linalool but not nerolidol (or the derived DMNT) upon JA application.

Volatile formation in plants is also effected by abiotic factors. Abscisic acid (ABA) seems to play a role in this regulation. Upon application of exogenous ABA to tomato the level of volatile compounds such as 1‐penten‐3‐one, β‐damascenone and benzaldehyde increased just as the expression of genes possibly involved in the biosynthesis of these volatiles, such as a lipoxygenase, alcohol dehydrogenase, carotenoid cleavage dioxygenase and a hydroperoxide lyase (Wu *et al*., 2018) (Figure 1). Promoter analysis showed that *cis*‐acting elements involved in ABA responsiveness (ABREs) exist in some of these genes. In a very different study, a similar phenomenon was demonstrated. Drought increased the level of ABA in *Salvia dolomitica* and increased the production of sesquiterpenes and the expression of FDP synthase that produces the substrate for sesquiterpene biosynthesis (Caser *et al*., 2019). In a study on the influence of salt on volatile emission from tomato leaves, however, the results are less clear (Zhang *et al*., 2018b). The production of some volatiles is altered, as is the expression of some genes involved in volatile biosynthesis, but the relationship between the two is unclear. Clearly, we are making ample progress in identifying the transcription factors regulating VOC biosynthetic pathways. The networks in which these TFs operate remain to be elucidated as well as the role of sRNAs and epigenetic regulation, especially in the developmental regulation of floral volatile biosynthesis.

## Emission of VOCs

Work on transport of volatiles intensified some 5 years ago when it was realized that volatiles, just as other lipophilic secondary metabolites, are likely to need active transport to be emitted by plants (Widhalm *et al*., 2015). Although there are still many caveats in our knowledge on transport (Tissier *et al*., 2017), there is also progress with the identification of the role of ABC transporters and LTP proteins as important players (Wang *et al*., 2016; Adebesin *et al*., 2017). Direct proof for the involvement of an ABC transporter in volatile emission in *Petunia hybrida* came from the silencing of *PhABCG1*, which resulted in a decrease in volatile emission (Adebesin *et al*., 2017). For the further unravelling of transport, particularly transcriptomics studies will be instrumental (Tissier *et al*., 2017). Intriguingly, in such a transcriptomics study in *Petunia axillaris*, an ABC transporter, PaABCG1, was reported with high homology to PhABCG1 (Amano *et al*., 2018). The authors did not carry out any functional characterization but discussed the fact that these types of ABCG transporters are mostly involved in transport of suberin and cuticle components. This also holds to some extent for the lipid transfer proteins for which Van der Krol and co‐workers show evidence that they also play a role – in concert with an ABC transporter for transport across the cell membrane – in transport of volatile and non‐volatile sesquiterpenoids across the cell wall (Wang *et al*., 2016). To what extent ABC transporters and LTPs play a role in the secretion of volatiles form the glandular cells of a trichome to the extracellular cavity remains to be investigated. A recent, elegant, opinion paper describes various scenarios on how this can be achieved (Tissier *et al*., 2017), but little information is known about this. This also holds true for how these volatiles are retained in the extracellular cavity as emission is often limited. Clearly, several questions remain and further studies are indeed needed to completely resolve the mystery of volatile transport and storage in plants (Tissier *et al*., 2017).

## Agricultural Importance of VOCs

The multitude of biological effects of VOCs makes them an attractive target to try to improve agriculture but the complexity of these interactions also make the application a large challenge (Beck *et al*., 2018). Beck and co‐authors review a number of possible applications in agricultural of our knowledge about volatiles. This includes the identification and subsequent application in the field of microbes that enhance flower volatile attractiveness to pollinators (Beck *et al*., 2018). Also they discussed the importance of taking the microbiome, in a broader sense, into account when studying the role of volatiles in plant−organism communication and its possible application in agriculture.

One of the applications that is being pursued for quite some time already is insect‐pest control. During the domestication of crops and production on a large scale, the emphasis of breeders historically was not on herbivore protection, but rather on optimizing traits such as yield and productivity. To compensate for this, (synthetic) pesticides were employed to allow food production on the massive scale needed. These synthetic compounds were often inspired by plant‐specialized metabolites but were chemically optimized for effectivity and stability. However, the use of synthetic chemicals for protection of our food is increasingly debated and the use of certain, very commonly used products such as neonicotinoids, have been severely restricted in the EU [regulations (EU) 2018/783‐784‐7885]. As an alternative to the use of synthetic pesticides, scientists and companies have turned to the introduction of insect resistance through genetic modification (Gutensohn *et al*., 2014; Sun *et al*., 2017; Douglas, 2018) and the employment of so‐called biopesticides (Pavela, 2016). A plethora of natural specialized metabolites is described in the literature for their potential in replacing chemicals. There are, however, only a handful of examples of such endogenously produced metabolites validated *in planta*, let alone in a field situation (Isman and Grieneisen, 2014; Kortbeek *et al*., 2018).

Arthropod pests will almost always evolve resistance to insecticides, which on average takes only 60–78 generations (Brevik *et al*., 2018). Different approaches, or layers of defence will therefore have to be combined to move towards a more sustainable solution. The use of semiochemicals and volatile signalling between plants and pest organism can be a part of such an integrated, more holistic and ecological, pest management approach (Beck *et al*., 2018). An intriguing example of the integration of semiochemicals in agriculture is the control of stemborers through a ‘push−pull’ strategy (Pickett and Khan, 2016). Here, cereals were offered protection from the stemborer moth, *Busseola fusca*, by the intercropped *Desmodium* spp. that provide ‘the push’ through producing volatiles that repel the moth. ‘The pull’ comes from (*E*)‐β‐caryophyllene emitted by Napier grass that attracts the moth and moreover produces a viscous substance that kills the larvae of the stemborer (Khan *et al*., 2007). A variation on the push−pull system described above is a semiochemical‐based pest management strategy that targets insect behaviour by using pheromones in a lure application (Mafra‐Neto *et al*., 2013). The authors developed a matrix that provide a steady release of a variety of volatile compounds including attractants such as sex pheromones in combination with insecticides, but also repellents. The system is targeted to disrupt mating, repel or attract‐and‐kill insects. The authors provide successful examples of the latter for serious crop‐pest species such as the fall armyworm (*Spodoptera fugiperda*) and the tomato pest, *Tuta absoluta*, both of which are lured to formula‐treated host plants by insect‐specific pheromones where they are killed by the insecticide. A formulation that releases verbenone, a repellent of the pine bark beetle *Dendroctonus ponderosae*, was shown to effectively keep the tree free of this bark beetle (Mafra‐Neto *et al*., 2013). The same holds for dimethyl disulfide, a repellent of the Asian citrus psyllid (*Diaphorina citri*) that transmits the Greening disease Huanglongbing (HLB), which severely threatens citrus production around the world (Mafra‐Neto *et al*., 2013). As described above, the ubiquitous volatile terpene (*E*)‐β‐caryophyllene repels a number of agriculturally important pests, including psyllids (Zhang *et al*., 2017). Intercropping (with the (*E*)‐β‐caryophyllene emitting guave) or possibly the application of (*E*)‐β‐caryophyllene dispensers may provide solutions for this enormous agricultural problem (Mafra‐Neto *et al*., 2013; Alquézar *et al*., 2017) (Figure 2f).

Insect control can potentially also be pursued using metabolic engineering of volatiles (Paul *et al*., 2016; Sun *et al*., 2016; Abbas *et al*., 2017; Tissier, 2018). In an attempt to change the resistance of chrysanthemum against aphids, Hu *et al*. (2018a) introduced chrysanthemol synthase that was isolated from pyrethrum and catalyzes the first committed step in pyrethrin biosynthesis, into chrysanthemum (Hu *et al*., 2018a) (Figure 1). Interestingly, this did not only result in the emission of the, expected, volatile chrysanthemol but also in accumulation of a chrysanthemol glycoside. Both compounds were shown to protect the transgenic chrysanthemum against aphids due to their repellent and antifeedant activity, respectively. In an attempt to protect citrus against *D. citri*, the Arabidopsis (*E*)‐β‐caryophyllene synthase was overexpressed in Arabidopsis (Alquézar *et al*., 2017). Transgenic plants with enhanced (*E*)‐β‐caryophyllene emission repelled *D. citri* suggesting that this strategy can potentially be used to protect citrus trees against HLB (Figure 2f).

Also the engineering of precursor pathways is an interesting tool to alter volatile production. Intriguing work of Dudareva and co‐workers (Henry *et al*., 2018) demonstrates an alternative role for the Nudix hydrolases that were previously shown to catalyze the first step in the formation of geraniol in rose (Magnard *et al*., 2015). They make it likely that the Arabidopsis NUDIX1 and 3 are involved in the dephosphorylation of isopentenyl diphosphate (IPP) to isopentenyl phosphate (IP), which then serves as a substrate for isopentenyl phosphate kinase. Although not completely understood yet IPP dephosphorylation seems to be an important regulatory mechanism as *nudix* mutants produce more terpenes while *AtNUDIX1* overexpression in tobacco results in lower volatile terpenoid production. Counter‐intuitively, however, when IP levels were increased by overexpression in tobacco of a bacterial phosphomevalonate decarboxylase (MPD), monoterpene and sesquiterpene production increased. Moreover, the authors then tested overexpression of Arabidopsis 3‐hydroxy‐3‐methylglutaryl‐coenzyme A reductase, *AtHMGR* – considered the rate‐limiting step in the mevalonate pathway – with *MPD* in tobacco and found a considerable further increase in both monoterpene and sesquiterpene production. Overexpression of these two genes in combination with the *Santalum album* santalene synthase resulted in almost 10‐fold higher emission of α‐santalene.

Zhang *et al*. (2018c) studied the effect of overexpression of two enzymes that catalyze an important, possibly rate‐limiting, steps in the plastidic MEP pathway, 1‐deoxy‐d‐xylulose‐5‐phosphate synthase (DXS) and 1‐deoxy‐d‐xylulose‐5‐phosphate reductoisomerase (DXR) (Zhang *et al*., 2018c). The encoding genes were isolated from *Lilium* ‘Siberia’ and heterologously overexpressed in tobacco of which the flowers produced more of the monoterpene, linalool (LiDXR overexpression line; as anticipated) but also of the sesquiterpene, caryophyllene (in both the LiDXR and LiDXS overexpression line), possibly as a result of increased IPP transport from the plastids to the cytosol as a result of the larger IPP pool produced in the transgenic lines (Figure 1).

In case the engineered volatiles needed to be stored in the plant rather than emitted, Delatte and colleagues invented an interesting engineering strategy in which oil bodies are heterologously introduced through transient expression of a diacylglycerol acyltransferase (*DGAT*), the transcription factor WRINKL1 and oleosin (*OLE1*) in *Nicotiana benthamiana* (Delatte *et al*., 2018). This indeed resulted in the formation of oil bodies that were associated with the heterologous sesquiterpenes that were simultaneously produced through the transient expression of sesquiterpene synthases, together resulting in a 17‐fold increased accumulation of volatile sesquiterpenes.

In conclusion, there are many promising scientific reports about the biological relevance of volatiles in the interaction of plants with other organisms. In these reports, the potential application of this knowledge in agriculture is usually mentioned. These possible applications include the use of volatiles in dispensers, or other matrices, the optimization of volatile production by plants through breeding or genetic modification and the application of selected microbes to change volatile emission by the plant. Particularly the use of natural enemies (that use volatile cues to find their prey), especially in greenhouses and the successful lure and kill strategies that employ attractive volatiles are examples that show that these approaches can work, despite the fact they have not really been optimized. The use of natural enemies for example has not, or hardly, been selected for despite the fact that scientists have shown there is genetic variation for HIPV induction in crops and that this results in difference in attractiveness of natural enemies (Kappers *et al*., 2011). Further research should show whether more intricate applications and optimization of the existing ones are possible such that they can be used to achieve a more sustainable agriculture.

## Conclusions/Future Directions

The field of VOC research is going through an exciting phase. We are seeing intriguing new developments such as the role of micro‐organisms in VOC communication, producing volatiles themselves (Lemfack *et al*., 2017; Schenkel *et al*., 2018), affecting the induction of HIPVs as endosymbionts of herbivorous insects (Frago *et al*., 2017) or changing the flower VOC blend because they colonize nectar (Vannette and Fukami, 2016; Rering *et al*., 2018). Also the elucidation of VOC transport, emission and accumulation and their perception in the receiver organism are hot topics in research and we are only just beginning to understand how specific volatiles and their blends convey information from one organism to the other and how flexible this process is. Advanced techniques such as genomics, transcriptomics and metabolomics will be associated with behavioural data using novel tools such as network analysis, large‐scale data integration and machine learning approaches, facilitating the interpretation of the biological meaning of the complex VOC blends in various dimensions. With these advanced approaches, the discovery of biosynthetic genes will also further speed up, allowing – through genetic modification/metabolic engineering in combination with behavioural assays – to further underpin the role of individual VOCs in the communication of plants with other organisms. This also holds for the genes and regulatory mechanisms underlying the regulation of the – constitutive and induced – formation of VOCs and their transport and sequestration. Genetic screens using the response to specific VOCs will be used to identify receptors and signal transduction cascades (Scala *et al*., 2017). With regard to the use of scientific knowledge on VOCs in agriculture, the use of natural enemies in greenhouses and lure and kill strategies are examples that already prove that the biological effect of VOCs can be employed in agriculture. A further deepening of our knowledge on the mechanisms underlying plant communication with beneficial as well as harmful organisms will surely provide the basis for more intricate applications in a more sustainable agriculture.

Box 1 **Open questions**

- How large is the role of the microbiome in determining the plant volatile blend?
- What is the underlying mechanism of (specificity in) the perception of volatiles by plants?
- What makes volatile communication between plants and other organisms an evolutionary stable strategy?

## Conflict of Interest

The authors declare that they have no conflict of interest.

## Author Contributions

All authors contributed equally to the writing and revising of the paper.

## References

[tpj14496-bib-0001] Aartsma, Y. , Bianchi, F.J. , Werf, W. , Poelman, E.H. and Dicke, M. (2017) Herbivore‐induced plant volatiles and tritrophic interactions across spatial scales. New Phytol. 216(4), 1054–1063. 28195346 10.1111/nph.14475 PMC6079636

[tpj14496-bib-0002] Abbas, F. , Ke, Y. , Yu, R. , Yue, Y. , Amanullah, S. , Jahangir, M.M. and Fan, Y. (2017) Volatile terpenoids: multiple functions, biosynthesis, modulation and manipulation by genetic engineering. Planta, 246(5), 803–816. 10.1007/s00425-017-2749-x. 28803364

[tpj14496-bib-0003] Adebesin, F. , Widhalm, J.R. , Boachon, B. ***et al.*** (2017) Emission of volatile organic compounds from *Petunia* flowers is facilitated by an ABC transporter. Science, 356(6345), 1386–1388. 10.1126/science.aan0826. 28663500

[tpj14496-bib-0004] Adebesin, F. , Widhalm, J.R. , Lynch, J.H. , McCoy, R.M. and Dudareva, N. (2018) A peroxisomal thioesterase plays auxiliary roles in plant β‐oxidative benzoic acid metabolism. Plant J. 93(5), 905–916. 10.1111/tpj.13818. 29315918

[tpj14496-bib-0005] Aguiar, J. , Roselino, A.C. , Sazima, M. and Giurfa, M. (2018) Can honey bees discriminate between floral‐fragrance isomers? J. Exp. Biol. 221(14), jeb180844 10.1242/jeb.180844 29798845

[tpj14496-bib-0006] Alquézar, B. , Volpe, H.X.L. , Magnani, R.F. , De Miranda, M.P. , Santos, M.A. , Wulff, N.A. , Bento, J.M.S. , Parra, J.R.P. , Bouwmeester, H. and Peña, L. (2017) β‐caryophyllene emitted from a transgenic Arabidopsis or chemical dispenser repels *Diaphorina citri*, vector of *Candidatus Liberibacters* . Sci. Rep. 7(1), 5639 10.1038/s41598-017-06119-w 28717202 PMC5514130

[tpj14496-bib-0007] Amano, I. , Kitajima, S. , Suzuki, H. , Koeduka, T. and Shitan, N. (2018) Transcriptome analysis of *Petunia axillaris* flowers reveals genes involved in morphological differentiation and metabolite transport. PLoS ONE, 13(6), e0198936 10.1371/journal.pone.0198936. 29902274 PMC6002047

[tpj14496-bib-0008] Ameye, M. , Allmann, S. , Verwaeren, J. , Smagghe, G. , Haesaert, G. , Schuurink, R.C. and Audenaert, K. (2018) Green leaf volatile production by plants: a meta‐analysis. New Phytol. 220(3), 666–683. 10.1111/nph.14671. 28665020

[tpj14496-bib-0009] Baldwin, I.T. and Schultz, J.C. (1983) Rapid changes in tree leaf chemistry induced by damage: evidence for communication between plants. Science, 221(4607), 277–279. 10.1126/science.221.4607.277. 17815197

[tpj14496-bib-0010] Beck, J.J. , Alborn, H.T. , Block, A.K. , Christensen, S.A. , Hunter, C.T. , Rering, C.C. , Seidl‐Adams, I. , Stuhl, C.J. , Torto, B. and Tumlinson, J.H. (2018) Interactions among plants, insects, and microbes: elucidation of inter‐organismal chemical communications in agricultural ecology. J. Agric. Food Chem. 66(26), 6663–6674. 29895142 10.1021/acs.jafc.8b01763

[tpj14496-bib-0011] Beyraghdar Kashkooli, A , Van der Krol, A and Bouwmeester, HJ (2018) Terpenoid biosynthesis in plants. In: Flavour Science, Proceedings of the 15th Weurman Flavour Research Symposium, 18‐22 September 2017. Verlag der Technischen Universität Graz, Graz, Austria.

[tpj14496-bib-0012] Bohman, B. , Karton, A. , Flematti, G.R. , Scaffidi, A. and Peakall, R. (2018a) Structure‐activity studies of semiochemicals from the spider orchid *Caladenia plicata* for sexual deception. J. Chem. Ecol. 44(5), 436–443. 10.1007/s10886-018-0946-0. 29549571

[tpj14496-bib-0013] Bohman, B. , Phillips, R.D. , Flematti, G.R. and Peakall, R. (2018b) (Methylthio)phenol semiochemicals are exploited by deceptive orchids as sexual attractants for *Campylothynnus* thynnine wasps. Fitoterapia, 126, 78–82. 10.1016/j.fitote.2017.09.022. 28965764

[tpj14496-bib-0014] Brevik, K. , Schoville, S.D. , Mota‐Sanchez, D. and Chen, Y.H. (2018) Pesticide durability and the evolution of resistance: a novel application of survival analysis. Pest Manag. Sci. 74(8), 1953–1963. 10.1002/ps.4899. 29493870

[tpj14496-bib-0015] Caser, M. , Chitarra, W. , D'Angiolillo, F. , Perrone, I. , Demasi, S. , Lovisolo, C. , Pistelli, L. , Pistelli, L. and Scariot, V. (2019) Drought stress adaptation modulates plant secondary metabolite production in *Salvia dolomitica* Codd. Ind. Crops Prod. 129, 85–96. 10.1016/j.indcrop.2018.11.068.

[tpj14496-bib-0016] Cheong, J.‐J. and Choi, Y.D. (2003) Methyl jasmonate as a vital substance in plants. Trends Genet. 19(7), 409–413. 10.1016/S0168-9525(03)00138-0. 12850447

[tpj14496-bib-0017] Clancy, M.V. , Zytynska, S.E. , Moritz, F. , Witting, M. , Schmitt‐Kopplin, P. , Weisser, W.W. and Schnitzler, J.P. (2018) Metabotype variation in a field population of tansy plants influences aphid host selection. Plant Cell Environ. 41(12), 2791–2805. 30035804 10.1111/pce.13407

[tpj14496-bib-0018] Cofer, T.M. , Engelberth, M. and Engelberth, J. (2018a) Green leaf volatiles protect maize (*Zea mays*) seedlings against damage from cold stress. Plant Cell Environ. 41(7), 1673–1682. 10.1111/pce.13204. 29601632

[tpj14496-bib-0019] Cofer, T.M. , Seidl‐Adams, I. and Tumlinson, J.H. (2018b) From acetoin to (*Z*)‐3‐hexen‐1‐ol: the diversity of volatile organic compounds that induce plant responses. J. Agric. Food Chem. 66(43), 11197–11208. 10.1021/acs.jafc.8b03010. 30293420

[tpj14496-bib-0020] Cuperlovic‐Culf, M. (2018) Machine learning methods for analysis of metabolic data and metabolic pathway modeling. Metabolites, 8(1), 4 10.3390/metabo8010004. PMC5875994 29324649

[tpj14496-bib-0021] Danner, H. , Brown, P. , Cator, E.A. , Harren, F.J. , van Dam, N.M. and Cristescu, S.M. (2015) Aboveground and belowground herbivores synergistically induce volatile organic sulfur compound emissions from shoots but not from roots. J. Chem. Ecol. 41(7), 631–640. 26195194 10.1007/s10886-015-0601-y PMC4525197

[tpj14496-bib-0022] Delatte, T.L. , Scaiola, G. , Molenaar, J. , de Sousa Farias, K. , Alves Gomes Albertti, L. , Busscher, J. , Verstappen, F. , Carollo, C. , Bouwmeester, H. and Beekwilder, J. (2018) Engineering storage capacity for volatile sesquiterpenes in *Nicotiana benthamiana* leaves. Plant Biotechnol. J. 16(12), 1997–2006. 10.1111/pbi.12933. 29682901 PMC6230952

[tpj14496-bib-0023] Douglas, A.E. (2018) Strategies for enhanced crop resistance to insect pests. Annu. Rev. Plant Biol. 69, 637–660. 29144774 10.1146/annurev-arplant-042817-040248

[tpj14496-bib-0024] Du Plessis, M. , Johnson, S.D. , Nicolson, S.W. , Bruyns, P.V. and Shuttleworth, A. (2018) Pollination of the “carrion flowers” of an African stapeliad (*Ceropegia mixta*: Apocynaceae): the importance of visual and scent traits for the attraction of flies. Plant Syst. Evol. 304(3), 357–372. 10.1007/s00606-017-1481-0.

[tpj14496-bib-0025] Duran‐Flores, D. and Heil, M. (2016) Sources of specificity in plant damaged‐self recognition. Curr. Opin. Plant Biol. 32, 77–87. 10.1016/j.pbi.2016.06.019. 27421107

[tpj14496-bib-0026] Engelberth, J. and Engelberth, M. (2019) The costs of green leaf volatile‐induced defense priming: temporal diversity in growth responses to mechanical wounding and insect herbivory. Plants, 8(1), 23. 10.3390/plants8010023 PMC6358849 30669247

[tpj14496-bib-0027] Erb, M. (2018) Volatiles as inducers and suppressors of plant defense and immunity‐origins, specificity, perception and signaling. Curr. Opin. Plant Biol. 44, 117–121. 10.1016/j.pbi.2018.03.008. 29674130

[tpj14496-bib-0028] Fan, R. , Chen, Y. , Ye, X. , Wu, J. , Lin, B. and Zhong, H. (2018) Transcriptome analysis of *Polianthes tuberosa* during floral scent formation. PLoS ONE, 13(9), e0199261 10.1371/journal.pone.0199261 30183703 PMC6124719

[tpj14496-bib-0029] Fincheira, P. and Quiroz, A. (2018) Microbial volatiles as plant growth inducers. Microbiol. Res. 208, 63–75. 10.1016/j.micres.2018.01.002. 29551213

[tpj14496-bib-0030] Frago, E. , Mala, M. , Weldegergis, B.T. , Yang, C. , McLean, A. , Godfray, H.C.J. , Gols, R. and Dicke, M. (2017) Symbionts protect aphids from parasitic wasps by attenuating herbivore‐induced plant volatiles. Nat. Commun. 8(1), 1860. 29192219 10.1038/s41467-017-01935-0 PMC5709398

[tpj14496-bib-0031] Fu, X. , Zhou, Y. , Zeng, L. , Dong, F. , Mei, X. , Liao, Y. , Watanabe, N. and Yang, Z. (2017) Analytical method for metabolites involved in biosynthesis of plant volatile compounds. RSC Advances, 7(31), 19363–19372. 10.1039/c7ra00766c.

[tpj14496-bib-0032] Gardner, E.M. , Gagne, R.J. , Kendra, P.E. , Montgomery, W.S. , Raguso, R.A. , McNeil, T.T. and Zerega, N.J.C. (2018) A flower in fruit's clothing: pollination of jackfruit (*Artocarpus heterophyllus*, Moraceae) by a new species of gall midge, *Clinodiplosis ultracrepidata* sp. nov. (Diptera: Cecidomyiidae). Int. J. Plant Sci. 179(5), 350–367. 10.1086/697115.

[tpj14496-bib-0033] Gasmi, L. , Martinez‐Solis, M. , Frattini, A. , Ye, M. , Collado, M.C. , Turlings, T.C.J. , Erb, M. and Herrero, S. (2018) Can herbivore‐induced volatiles protect plants by increasing the herbivores’ susceptibility to natural pathogens? Appl. Environ. Microbiol. 85, e01468–18. 10.1128/aem.01468-18 30366995 PMC6293100

[tpj14496-bib-0034] Goossens, J. , Mertens, J. and Goossens, A. (2017) Role and functioning of bHLH transcription factors in jasmonate signalling. J. Exp. Bot. 68(6), 1333–1347. 10.1093/jxb/erw440. 27927998

[tpj14496-bib-0035] Gou, J.‐Y. , Felippes, F.F. , Liu, C.‐J. , Weigel, D. and Wang, J.‐W. (2011) Negative regulation of anthocyanin biosynthesis in Arabidopsis by a miR156‐targeted SPL transcription factor. Plant Cell, 23(4), 1512–1522. 21487097 10.1105/tpc.111.084525 PMC3101539

[tpj14496-bib-0036] Gutensohn, M. , Nguyen, T.T. , McMahon, R.D. III , Kaplan, I. , Pichersky, E. and Dudareva, N. (2014) Metabolic engineering of monoterpene biosynthesis in tomato fruits via introduction of the non‐canonical substrate neryl diphosphate. Metab. Eng. 24, 107–116. 24831707 10.1016/j.ymben.2014.05.008

[tpj14496-bib-0037] Hatano, E. , Saveer, A.M. , Borrero‐Echeverry, F. ***et al.*** (2015) A herbivore‐induced plant volatile interferes with host plant and mate location in moths through suppression of olfactory signalling pathways. BMC Biol. 13(1), 75 10.1186/s12915-015-0188-3. 26377197 PMC4571119

[tpj14496-bib-0038] Haugeneder, A. , Trinkl, J. , Härtl, K. , Hoffmann, T. , Allwood, J.W. and Schwab, W. (2018) Answering biological questions by analysis of the strawberry metabolome. Metabolomics, 14(11), 145 10.1007/s11306-018-1441-x. 30830391 PMC6394451

[tpj14496-bib-0039] Haverkamp, A. , Bing, J. , Badeke, E. , Hansson, B.S. and Knaden, M. (2016a) Innate olfactory preferences for flowers matching proboscis length ensure optimal energy gain in a hawkmoth. Nat. Commun. 7, 11644 10.1038/ncomms11644. 27173441 PMC4869250

[tpj14496-bib-0040] Haverkamp, A. , Yon, F. , Keesey, I.W. , Missbach, C. , Koenig, C. , Hansson, B.S. , Baldwin, I.T. , Knaden, M. and Kessler, D. (2016b) Hawkmoths evaluate scenting flowers with the tip of their proboscis. eLife, 5, e15039 10.7554/elife.15039. 27146894 PMC4884077

[tpj14496-bib-0041] Haverkamp, A. , Hansson, B.S. and Knaden, M. (2018) Combinatorial codes and labeled lines: how insects use olfactory cues to find and judge food, mates, and oviposition sites in complex environments. Front. Physiol. 9, 49 10.3389/fphys.2018.00049. 29449815 PMC5799900

[tpj14496-bib-0042] He, X. , Wang, H. , Yang, J. , Deng, K. , Wang, T. and Cheng, Z.M. (2018) RNA sequencing on *Amomum villosum* Lour. induced by MeJA identifies the genes of WRKY and terpene synthases involved in terpene biosynthesis. Genome, 61(2), 91–102. 10.1139/gen-2017-0142 29338341

[tpj14496-bib-0043] Heil, M. and Karban, R. (2010) Explaining evolution of plant communication by airborne signals. Trends Ecol. Evol. 25(3), 137–144. 10.1016/j.tree.2009.09.010. 19837476

[tpj14496-bib-0044] Helms, A.M. , De Moraes, C.M. , Tröger, A. , Alborn, H.T. , Francke, W. , Tooker, J.F. and Mescher, M.C. (2017) Identification of an insect‐produced olfactory cue that primes plant defenses. Nat. Commun. 8(1), 337 10.1038/s41467-017-00335-8. 28835618 PMC5569085

[tpj14496-bib-0045] Henry, L.K. , Thomas, S.T. , Widhalm, J.R. , Lynch, J.H. , Davis, T.C. , Kessler, S.A. , Bohlmann, J. , Noel, J.P. and Dudareva, N. (2018) Contribution of isopentenyl phosphate to plant terpenoid metabolism. Nat. Plants, 4(9), 721–729. 10.1038/s41477-018-0220-z. 30127411

[tpj14496-bib-0046] Holopainen, J.K. and Gershenzon, J. (2010) Multiple stress factors and the emission of plant VOCs. Trends Plant Sci. 15(3), 176–184. 20144557 10.1016/j.tplants.2010.01.006

[tpj14496-bib-0047] Hong, G.J. , Xue, X.Y. , Mao, Y.B. , Wang, L.J. and Chen, X.Y. (2012) Arabidopsis MYC2 interacts with DELLA proteins in regulating sesquiterpene synthase gene expression. Plant Cell, 24(6), 2635–2648. 10.1105/tpc.112.098749. 22669881 PMC3406894

[tpj14496-bib-0048] Houshyani, B. , Assareh, M. , Busquets, A. , Ferrer, A. , Bouwmeester, H.J. and Kappers, I.F. (2013) Three‐step pathway engineering results in more incidence rate and higher emission of nerolidol and improved attraction of *Diadegma semiclausum* . Metab. Eng. 15(1), 88–97. 10.1016/j.ymben.2012.10.002. 23154132

[tpj14496-bib-0049] Hu, H. , Li, J. , Delatte, T. , Vervoort, J. , Gao, L. , Verstappen, F. , Xiong, W. , Gan, J. , Jongsma, M.A. and Wang, C. (2018a) Modification of chrysanthemum odour and taste with chrysanthemol synthase induces strong dual resistance against cotton aphids. Plant Biotechnol. J. 16(8), 1434–1445. 10.1111/pbi.12885. 29331089 PMC6041446

[tpj14496-bib-0050] Hu, L. , Ye, M. and Erb, M. (2018b) Integration of two herbivore‐induced plant volatiles results in synergistic effects on plant defence and resistance. Plant Cell Environ. 42, 959–971. 10.1111/pce.13443. 30195252 PMC6392123

[tpj14496-bib-0051] Huang, M. , Fan, R. , Ye, X. , Lin, R. , Luo, Y. , Fang, N. , Zhong, H. and Chen, S. (2018) The transcriptome of flower development provides insight into floral scent formation in *Freesia hybrida* . Plant Growth Regul. 86(1), 93–104. 10.1007/s10725-018-0413-5.

[tpj14496-bib-0052] Isman, M.B. and Grieneisen, M.L. (2014) Botanical insecticide research: many publications, limited useful data. Trends Plant Sci. 19(3), 140–145. 24332226 10.1016/j.tplants.2013.11.005

[tpj14496-bib-0053] Johnson, S.D. and Schiestl, F.P. (2016) Floral Mimicry. Oxford: Oxford University Press.

[tpj14496-bib-0054] Joshi, J.R. , Khazanov, N. , Senderowitz, H. , Burdman, S. , Lipsky, A. and Yedidia, I. (2016) Plant phenolic volatiles inhibit quorum sensing in pectobacteria and reduce their virulence by potential binding to ExpI and ExpR proteins. Sci. Rep. 6, 38126. 27905512 10.1038/srep38126 PMC5131480

[tpj14496-bib-0055] Junker, R.R. (2018) A biosynthetically informed distance measure to compare secondary metabolite profiles. Chemoecology, 28(1), 29–37. 10.1007/s00049-017-0250-4. 29540963 PMC5840250

[tpj14496-bib-0056] Jürgens, A. , Wee, S.L. , Shuttleworth, A. and Johnson, S.D. (2013) Chemical mimicry of insect oviposition sites: a global analysis of convergence in angiosperms. Ecol. Lett. 16(9), 1157–1167. 10.1111/ele.12152. 23841830

[tpj14496-bib-0057] Kant, M.R. , Jonckheere, W. , Knegt, B. ***et al.*** (2015) Mechanisms and ecological consequences of plant defence induction and suppression in herbivore communities. Ann. Bot. 115(7), 1015–1051. 10.1093/aob/mcv054. 26019168 PMC4648464

[tpj14496-bib-0058] Kappers, I.F. , Hoogerbrugge, H. , Bouwmeester, H.J. and Dicke, M. (2011) Variation in herbivory‐induced volatiles among cucumber (*Cucumis sativus* L.) varieties has consequences for the attraction of carnivorous natural enemies. J. Chem. Ecol. 37(2), 150–160. 21249432 10.1007/s10886-011-9906-7 PMC3043237

[tpj14496-bib-0059] Karban, R. , Wetzel, W.C. , Shiojiri, K. , Ishizaki, S. , Ramirez, S.R. and Blande, J.D. (2014a) Deciphering the language of plant communication: volatile chemotypes of sagebrush. New Phytol. 204(2), 380–385. 10.1111/nph.12887. 24920243

[tpj14496-bib-0060] Karban, R. , Yang, L.H. and Edwards, K.F. (2014b) Volatile communication between plants that affects herbivory: a meta‐analysis. Ecol. Lett. 17(1), 44–52. 10.1111/ele.12205. 24165497

[tpj14496-bib-0061] Karban, R. , Wetzel, W.C. , Shiojiri, K. , Pezzola, E. and Blande, J.D. (2016) Geographic dialects in volatile communication between sagebrush individuals. Ecology, 97(11), 2917–2924. 10.1002/ecy.1573. 27870040

[tpj14496-bib-0062] Kessler, A. and Kalske, A. (2018) Plant secondary metabolite diversity and species interactions. Annu. Rev. Ecol. Evol. Syst. 49, 115–138.

[tpj14496-bib-0300] Khan, Z.R. , Midega, C.A.O. , Wadhams, L.J. , Pickett, J.A. and Mumuni, A. (2007) Evaluation of Napier grass (*Pennisetum purpureum*) varieties for use as trap plants for the management of African stemborer (*Busseola fusca*) in a push-pull strategy. Entomol. Exp. Appl. 124, 201–211.

[tpj14496-bib-0063] Knauer, A.C. , Bakhtiari, M. and Schiestl, F.P. (2018) Crab spiders impact floral‐signal evolution indirectly through removal of florivores. Nat. Commun. 9, 1367 10.1038/s41467-018-03792-x. 29636464 PMC5893632

[tpj14496-bib-0064] Kortbeek, R.W. , van der Gragt, M. and Bleeker, P.M. (2018) Endogenous plant metabolites against insects. Eur. J. Plant Pathol. 54, 67–90.

[tpj14496-bib-0065] Kumar, Y. , Khan, F. , Rastogi, S. and Shasany, A.K. (2018) Genome‐wide detection of terpene synthase genes in holy basil (*Ocimum sanctum* L.). PLoS ONE, 13, (11) 10.1371/journal.pone.0207097. PMC6239295 30444870

[tpj14496-bib-0066] Lackus, N.D. , Lackner, S. , Gershenzon, J. , Unsicker, S.B. and Köllner, T.G. (2018) The occurrence and formation of monoterpenes in herbivore‐damaged poplar roots. Sci. Rep. 8(1), 17936 10.1038/s41598-018-36302-6. 30560919 PMC6299004

[tpj14496-bib-0067] Lee, S. , Badieyan, S. , Bevan, D.R. , Herde, M. , Gatz, C. and Tholl, D. (2010) Herbivore‐induced and floral homoterpene volatiles are biosynthesized by a single P450 enzyme (CYP82G1) in Arabidopsis. Proc. Natl. Acad. Sci. USA, 107(49), 21205–21210. 10.1073/pnas.1009975107. 21088219 PMC3000306

[tpj14496-bib-0068] Lemfack, M.C. , Gohlke, B.‐O. , Toguem, S.M.T. , Preissner, S. , Piechulla, B. and Preissner, R. (2017) mVOC 2.0: a database of microbial volatiles. Nucleic Acids Res. 46(D1), D1261–D1265. 10.1093/nar/gkx1016 PMC5753297 29106611

[tpj14496-bib-0069] Li, C. , Schilmiller, A.L. , Liu, G. ***et al.*** (2005) Role of β‐oxidation in jasmonate biosynthesis and systemic wound signaling in tomato. Plant Cell, 17(3), 971–986. 10.1105/tpc.104.029108. 15722469 PMC1069712

[tpj14496-bib-0070] Li, S. , Wang, H. , Li, F. , Chen, Z. , Li, X. , Zhu, L. , Wang, G. , Yu, J. , Huang, D. and Lang, Z. (2015) The maize transcription factor EREB58 mediates the jasmonate‐induced production of sesquiterpene volatiles. Plant J. 84(2), 296–308. 10.1111/tpj.12994. 26303437

[tpj14496-bib-0071] Li, X. , Xu, Y. , Shen, S. , Yin, X. , Klee, H. , Zhang, B. , Chen, K. and Hancock, R. (2017) Transcription factor CitERF71 activates the terpene synthase gene CitTPS16 involved in the synthesis of *E*‐geraniol in sweet orange fruit. J. Exp. Bot. 68(17), 4929–4938. 10.1093/jxb/erx316. 28992329 PMC5853461

[tpj14496-bib-0072] Liang, M.H. , Zhu, J. and Jiang, J.G. (2018) Carotenoids biosynthesis and cleavage related genes from bacteria to plants. Crit. Rev. Food Sci. Nutr. 58(14), 2314–2333. 10.1080/10408398.2017.1322552. 28609133

[tpj14496-bib-0073] Liu, D. , Huang, X. , Jing, W. , An, X. , Zhang, Q. , Zhang, H. , Zhou, J. , Zhang, Y. and Guo, Y. (2018a) Identification and functional analysis of two P450 enzymes of *Gossypium hirsutum* involved in DMNT and TMTT biosynthesis. Plant Biotechnol. J. 16(2), 581–590. 10.1111/pbi.12797. 28710782 PMC5787835

[tpj14496-bib-0074] Liu, G.F. , Liu, J.J. , He, Z.R. , Wang, F.M. , Yang, H. , Yan, Y.F. , Gao, M.J. , Gruber, M.Y. , Wan, X.C. and Wei, S. (2018b) Implementation of CsLIS/NES in linalool biosynthesis involves transcript splicing regulation in *Camellia sinensis* . Plant Cell Environ. 41(1), 176–186. 10.1111/pce.13080. 28963730

[tpj14496-bib-0075] López‐Gresa, M.P. , Payá, C. , Ozáez, M. , Rodrigo, I. , Conejero, V. , Klee, H. , Bellés, J.M. and Lisón, P. (2018) A new role for green leaf volatile esters in tomato stomatal defense against *Pseudomonas syringe* pv. Tomato. Front. Plant Sci. 9, 1855 10.3389/fpls.2018.01855. 30619420 PMC6305539

[tpj14496-bib-0076] Mafra‐Neto, A. , Frédérique, M. , Fettig, C.J. , Munson, A.S. , Perring, T.M. , Stelinski, L.L. , Stoltman, L. , Mafra, L.E. , Borges, R. and Vargas, R.I. (2013) Manipulation of insect behavior with Specialized Pheromone & Lure Application Technology (SPLAT^®^) In Natural Products for Pest Management (Rimando A.M. and Duke S.O., eds). Chapter 4. Washington, DC: ACS Publications, pp. 31–58.

[tpj14496-bib-0077] Magnard, J.L. , Roccia, A. , Caissard, J.C. ***et al.*** (2015) Biosynthesis of monoterpene scent compounds in roses. Science, 349(6243), 81–83. 10.1126/science.aab0696. 26138978

[tpj14496-bib-0078] Markovic, D. , Colzi, I. , Taiti, C. , Ray, S. , Scalone, R. , Gregory Ali, J. , Mancuso, S. and Ninkovic, V. (2018) Airborne signals synchronize the defenses of neighboring plants in response to touch. J. Exp. Bot. 70, 691–700. 10.1093/jxb/ery375. PMC6322579 30380091

[tpj14496-bib-0079] Martin, K.R. , More, M. , Hipolito, J. , Charlemagne, S. , Schlumpberger, B.O. and Raguso, R.A. (2017) Spatial and temporal variation in volatile composition suggests olfactory division of labor within the trap flowers of *Aristolochia gigantea* . Flora, 232, 153–168. 10.1016/j.flora.2016.09.005.

[tpj14496-bib-0080] Martínez‐Medina, A. , Van Wees, S.C. and Pieterse, C.M. (2017) Airborne signals from Trichoderma fungi stimulate iron uptake responses in roots resulting in priming of jasmonic acid‐dependent defences in shoots of Arabidopsis thaliana and Solanum lycopersicum. Plant Cell Environ. 40(11), 2691–2705. 28667819 10.1111/pce.13016

[tpj14496-bib-0081] Massalha, H. , Korenblum, E. , Tholl, D. and Aharoni, A. (2017) Small molecules below‐ground: the role of specialized metabolites in the rhizosphere. Plant J. 90(4), 788–807. 28333395 10.1111/tpj.13543

[tpj14496-bib-0082] McCormick, A.C. , Unsicker, S.B. and Gershenzon, J. (2012) The specificity of herbivore‐induced plant volatiles in attracting herbivore enemies. Trends Plant Sci. 17(5), 303–310. 22503606 10.1016/j.tplants.2012.03.012

[tpj14496-bib-0083] Milet‐Pinheiro, P. , Silva, J.B.F. , Navarro, D. , Machado, I.C.S. and Gerlach, G. (2018) Notes on pollination ecology and floral scent chemistry of the rare neotropical orchid *Catasetum galeritum* Rchb.f. Plant Species Biol. 33(2), 158–163. 10.1111/1442-1984.12202

[tpj14496-bib-0084] Moreira, X. , Nell, C.S. , Katsanis, A. , Rasmann, S. and Mooney, K.A. (2018) Herbivore specificity and the chemical basis of plant‐plant communication in *Baccharis salicifolia* (Asteraceae). New Phytol. 220(3), 703–713. 10.1111/nph.14164. 27597176

[tpj14496-bib-0085] Nagashima, A. , Higaki, T. , Koeduka, T. , Ishigami, K. , Hosokawa, S. , Watanabe, H. , Matsui, K. , Hasezawa, S. and Touhara, K. (2018) Transcriptional regulators involved in responses to volatile organic compounds in plants. J. Biol. Chem. 294, 2256–2266. 10.1074/jbc.RA118.005843. 30593507 PMC6378981

[tpj14496-bib-0086] Nieuwenhuizen, N.J. , Chen, X. , Wang, M.Y. , Matich, A.J. , Perez, R.L. , Allan, A.C. , Green, S.A. and Atkinson, R.G. (2015) Natural variation in monoterpene synthesis in kiwifruit: transcriptional regulation of terpene synthases by NAC and ETHYLENE‐INSENSITIVE3‐like transcription factors. Plant Physiol. 167(4), 1243–1258. 10.1104/pp.114.254367. 25649633 PMC4378164

[tpj14496-bib-0087] Park, S.W. , Kaimoyo, E. , Kumar, D. , Mosher, S. and Klessig, D.F. (2007) Methyl salicylate is a critical mobile signal for plant systemic acquired resistance. Science, 318(5847), 113–116. 10.1126/science.1147113. 17916738

[tpj14496-bib-0088] Paul, I. , Bhadoria, P.S. and Mitra, A. (2016) Plant volatile genomics: recent developments and putative applications in agriculture. Recent Pat. Biotechnol. 10(1), 4–11. 10.2174/1872208310666160908144935. 27634358

[tpj14496-bib-0089] Pauwels, L. , Barbero, G.F. , Geerinck, J. ***et al.*** (2010) NINJA connects the co‐repressor TOPLESS to jasmonate signalling. Nature, 464(7289), 788–791. 10.1038/nature08854. 20360743 PMC2849182

[tpj14496-bib-0090] Pavela, R. (2016) History, presence and perspective of using plant extracts as commercial botanical insecticides and farm products for protection against insects–a review. Plant Protect. Sci. 52(4), 229–241.

[tpj14496-bib-0091] Perez‐Cembranos, A. , Perez‐Mellado, V. and Cooper, W.E. (2018) Balearic lizards use chemical cues from a complex deceptive mimicry to capture attracted pollinators. Ethology, 124(4), 260–268. 10.1111/eth.12728.

[tpj14496-bib-0092] Pichersky, E. and Raguso, R.A. (2018) Why do plants produce so many terpenoid compounds? New Phytol. 220(3), 692–702. 10.1111/nph.14178. 27604856

[tpj14496-bib-0093] Pickett, J.A. and Khan, Z.R. (2016) Plant volatile‐mediated signalling and its application in agriculture: successes and challenges. New Phytol. 212(4), 856–870. 27874990 10.1111/nph.14274

[tpj14496-bib-0094] Piechulla, B. , Lemfack, M.C. and Kai, M. (2017) Effects of discrete bioactive microbial volatiles on plants and fungi. Plant Cell Environ. 40(10), 2042–2067. 28643880 10.1111/pce.13011

[tpj14496-bib-0095] Qualley, A.V. , Widhalm, J.R. , Adebesin, F. , Kish, C.M. and Dudareva, N. (2012) Completion of the core beta‐oxidative pathway of benzoic acid biosynthesis in plants. Proc. Natl Acad. Sci. USA, 109(40), 16383–16388. 10.1073/pnas.1211001109. 22988098 PMC3479573

[tpj14496-bib-0096] Raguso, R.A. (2008) Wake up and smell the roses: the ecology and evolution of floral scent. Annu. Rev. Ecol. Evol. Syst. 39, 549–569. 10.1146/annurev.ecolsys.38.091206.095601.

[tpj14496-bib-0097] Ramya, M. , Kwon, O.K. , An, H.R. , Park, P.M. , Baek, Y.S. and Park, P.H. (2017) Floral scent: regulation and role of MYB transcription factors. Phytochem. Lett. 19, 114–120. 10.1016/j.phytol.2016.12.015.

[tpj14496-bib-0098] Rering, C.C. , Beck, J.J. , Hall, G.W. , McCartney, M.M. and Vannette, R.L. (2018) Nectar‐inhabiting microorganisms influence nectar volatile composition and attractiveness to a generalist pollinator. New Phytol. 220(3), 750–759. 10.1111/nph.14809. 28960308

[tpj14496-bib-0099] Rhoades, D.F. (1983) Responses of Alder and Willow to attack by tent caterpillars and webworms: evidence for pheromonal sensitivity of willows In Plant Resistance to Insects (Hedin P.A. ed.), vol 208, ACS Symposium Series. Washington, DC: ACS Publications, pp. 55–68. 10.1021/bk-1983-0208.ch004

[tpj14496-bib-0100] Richter, A. , Schaff, C. , Zhang, Z.W. ***et al.*** (2016) Characterization of biosynthetic pathways for the production of the volatile homoterpenes DMNT and TMTT in *Zea mays* . Plant Cell, 28(10), 2651–2665. 10.1105/tpc.15.00919. 27662898 PMC5134970

[tpj14496-bib-0101] Riedlmeier, M. , Ghirardo, A. , Wenig, M. , Knappe, C. , Koch, K. , Georgii, E. , Dey, S. , Parker, J.E. , Schnitzler, J.‐P. and Vlot, C. (2017) Monoterpenes support systemic acquired resistance within and between plants. Plant Cell, 29, 1440–1459. 28536145 10.1105/tpc.16.00898 PMC5502447

[tpj14496-bib-0102] Ryu, C.M. , Farag, M.A. , Hu, C.H. , Reddy, M.S. , Wei, H.X. , Pare, P.W. and Kloepper, J.W. (2003) Bacterial volatiles promote growth in Arabidopsis. Proc. Natl Acad. Sci. USA, 100(8), 4927–4932. 10.1073/pnas.0730845100. 12684534 PMC153657

[tpj14496-bib-0103] Santos‐Sánchez, N.F. , Salas‐Coronado, R. , Hernández‐Carlos, B. and Villanueva‐Cañongo, C. (2019) Shikimic acid pathway in biosynthesis of phenolic compounds. In: Plant Physiological Aspects of Phenolic Compounds. IntechOpen. 10.5772/intechopen.83815

[tpj14496-bib-0104] Scala, A. , Mirabella, R. , Goedhart, J. , de Vries, M. , Haring, M.A. and Schuurink, R.C. (2017) Forward genetic screens identify a role for the mitochondrial HER2 in E‐2‐hexenal responsiveness. Plant Mol. Biol. 95(4–5), 399–409. 10.1007/s11103-017-0659-8. 28918565 PMC5688203

[tpj14496-bib-0105] Schaller, G.E. and Bleecker, A.B. (1995) Ethylene‐binding sites generated in yeast expressing the Arabidopsis *ETR1* gene. Science, 270(5243), 1809–1811. 8525372 10.1126/science.270.5243.1809

[tpj14496-bib-0106] Schenkel, D. , Maciá‐Vicente, J.G. , Bissell, A. and Splivallo, R. (2018) Fungi indirectly affect plant root architecture by modulating soil volatile organic compounds. Front. Microbiol. 9, 1847. 30150975 10.3389/fmicb.2018.01847 PMC6099090

[tpj14496-bib-0107] Schiestl, F.P. (2015) Ecology and evolution of floral volatile‐mediated information transfer in plants. New Phytol. 206(2), 571–577. 10.1111/nph.13243. 25605223

[tpj14496-bib-0108] Schiestl, F.P. and Johnson, S.D. (2013) Pollinator‐mediated evolution of floral signals. Trends Ecol. Evol. 28(5), 307–315. 10.1016/j.tree.2013.01.019. 23480953

[tpj14496-bib-0109] Schwab, R. , Palatnik, J.F. , Riester, M. , Schommer, C. , Schmid, M. and Weigel, D. (2005) Specific effects of microRNAs on the plant transcriptome. Dev. Cell, 8(4), 517–527. 15809034 10.1016/j.devcel.2005.01.018

[tpj14496-bib-0110] Sharifi, R. and Ryu, C.M. (2018) Revisiting bacterial volatile‐mediated plant growth promotion: lessons from the past and objectives for the future. Ann. Bot. 122(3), 349–358. 10.1093/aob/mcy108. 29982345 PMC6110341

[tpj14496-bib-0111] Sharifi, R. , Lee, S.M. and Ryu, C.M. (2018) Microbe‐induced plant volatiles. New Phytol. 220(3), 684–691. 10.1111/nph.14955. 29266296

[tpj14496-bib-0112] Shikano, I. , Rosa, C. , Tan, C.‐W. and Felton, G.W. (2017) Tritrophic interactions: microbe‐mediated plant effects on insect herbivores. Annu. Rev. Phytopathol. 55, 313–331. 28590879 10.1146/annurev-phyto-080516-035319

[tpj14496-bib-0113] Singh, N. and Sharma, A. (2017) Turmeric (*Curcuma longa*): miRNAs and their regulating targets are involved in development and secondary metabolite pathways. C. R. Biol. 340(11–12), 481–491. 29126713 10.1016/j.crvi.2017.09.009

[tpj14496-bib-0114] Sørensen, M. , Neilson, E.H.J. and Møller, B.L. (2018) Oximes: unrecognized chameleons in general and specialized plant metabolism. Mol. Plant, 11(1), 95–117. 10.1016/j.molp.2017.12.014. 29275165

[tpj14496-bib-0115] Stahl, E. , Hilfiker, O. and Reymond, P. (2018) Plant–arthropod interactions: who is the winner? Plant J. 93(4), 703–728. 10.1111/tpj.13773. 29160609

[tpj14496-bib-0116] Strutz, A. , Soelter, J. , Baschwitz, A. , Farhan, A. , Grabe, V. , Rybak, J. , Knaden, M. , Schmuker, M. , Hansson, B.S. and Sachse, S. (2014) Decoding odor quality and intensity in the Drosophila brain. eLife, 3, 21 10.7554/eLife.04147. PMC4270039 25512254

[tpj14496-bib-0117] Sugimoto, K. , Matsui, K. , Iijima, Y. ***et al.*** (2014) Intake and transformation to a glycoside of (*Z*)‐3‐hexenol from infested neighbors reveals a mode of plant odor reception and defense. Proc. Natl Acad. Sci. USA, 111(19), 7144–7149. 10.1073/pnas.1320660111. 24778218 PMC4024874

[tpj14496-bib-0118] Sugimoto, K. , Matsui, K. and Takabayashi, J. (2015) Conversion of volatile alcohols into their glucosides in Arabidopsis. Commun. Integr. Biol. 8(1), e992731 10.4161/19420889.2014.992731. 26629260 PMC4594374

[tpj14496-bib-0119] Sun, P. , Schuurink, R.C. , Caissard, J.C. , Hugueney, P. and Baudino, S. (2016) My way: noncanonical biosynthesis pathways for plant volatiles. Trends Plant Sci. 21(10), 884–894. 10.1016/j.tplants.2016.07.007. 27475252

[tpj14496-bib-0120] Sun, Y. , Huang, X. , Ning, Y. , Jing, W. , Bruce, T.J. , Qi, F. , Xu, Q. , Wu, K. , Zhang, Y. and Guo, Y. (2017) TPS46, a rice terpene synthase conferring natural resistance to bird cherry‐oat aphid, *Rhopalosiphum padi* (Linnaeus). Front. Plant Sci. 8, 110. 28217135 10.3389/fpls.2017.00110 PMC5289981

[tpj14496-bib-0121] Teichert, H. , Dötterl, S. and Gottsberger, G. (2018) Scent emissions and floral nutrients of Carludovicoideae (Cyclanthaceae) and their importance for associated beetles. Plant Syst. Evol. 304(7), 831–839. 10.1007/s00606-018-1513-4.

[tpj14496-bib-0122] Tian, J.P. , Ma, Z.Y. , Zhao, K.G. , Zhang, J. , Xiang, L. and Chen, L.Q. (2018) Transcriptomic and proteomic approaches to explore the differences in monoterpene and benzenoid biosynthesis between scented and unscented genotypes of wintersweet. Physiol. Plant. 166, 478–493. 10.1111/ppl.12828 30216458

[tpj14496-bib-0123] Tissier, A. (2018) Harnessing plant trichome biochemistry for the production of useful compounds In Molecular Pharming: Applications, Challenges and Emerging Areas (Kermode A.R. and Jiang L., eds.). Wiley Blackwell, pp. 353–382. 10.1002/9781118801512.ch14

[tpj14496-bib-0124] Tissier, A. , Morgan, J.A. and Dudareva, N. (2017) Plant Volatiles: going ‘In’ but not ‘Out’ of Trichome Cavities. Trends Plant Sci. 22(11), 930–938. 10.1016/j.tplants.2017.09.001. 28958712

[tpj14496-bib-0125] Tungadi, T. , Groen, S.C. , Murphy, A.M. , Pate, A.E. , Iqbal, J. , Bruce, T.J. , Cunniffe, N.J. and Carr, J.P. (2017) Cucumber mosaic virus and its 2b protein alter emission of host volatile organic compounds but not aphid vector settling in tobacco. Virol. J. 14(1), 91. 28468686 10.1186/s12985-017-0754-0 PMC5415739

[tpj14496-bib-0126] Turlings, T.C.J. and Erb, M. (2018) Tritrophic interactions mediated by herbivore‐induced plant volatiles: mechanisms, ecological relevance, and application potential. Annu. Rev. Entomol. 63(1), 433–452. 10.1146/annurev-ento-020117-043507. 29324043

[tpj14496-bib-0127] Van Dam, N.M. , Gossa, M.W. , Mathur, V. and Tytgat, T.O. (2018) Differences in hormonal signaling triggered by two root‐feeding nematode species result in contrasting effects on aphid population growth. Front. Ecol. Evol. 6, 88.

[tpj14496-bib-0128] Van der Niet, T. , Hansen, D.M. and Johnson, S.D. (2011) Carrion mimicry in a South African orchid: flowers attract a narrow subset of the fly assemblage on animal carcasses. Ann. Bot. 107(6), 981–992. 10.1093/aob/mcr048. 21402538 PMC3080630

[tpj14496-bib-0129] Vannette, R.L. and Fukami, T. (2016) Nectar microbes can reduce secondary metabolites in nectar and alter effects on nectar consumption by pollinators. Ecology, 97(6), 1410–1419. 10.1890/15-0858.1. 27459772

[tpj14496-bib-0130] Villarroel, C.A. , Jonckheere, W. , Alba, J.M. , Glas, J.J. , Dermauw, W. , Haring, M.A. , Van Leeuwen, T. , Schuurink, R.C. and Kant, M.R. (2016) Salivary proteins of spider mites suppress defenses in *Nicotiana benthamiana* and promote mite reproduction. Plant J. 86(2), 119–131. 10.1111/tpj.13152. 26946468

[tpj14496-bib-0131] Vos, I.A. , Verhage, A. , Schuurink, R.C. , Watt, L.G. , Pieterse, C.M. and Van Wees, S.C. (2013) Onset of herbivore‐induced resistance in systemic tissue primed for jasmonate‐dependent defenses is activated by abscisic acid. Front. Plant Sci. 4, 539 10.3389/fpls.2013.00539. 24416038 PMC3874679

[tpj14496-bib-0132] Wang, B. , Kashkooli, A.B. , Sallets, A. ***et al.*** (2016) Transient production of artemisinin in *Nicotiana benthamiana* is boosted by a specific lipid transfer protein from *A. annua* . Metab. Eng. 38, 159–169. 10.1016/j.ymben.2016.07.004. 27421621

[tpj14496-bib-0133] Wee, S.L. , Tan, S.B. and Jürgens, A. (2018) Pollinator specialization in the enigmatic *Rafflesia cantleyi*: a true carrion flower with species‐specific and sex‐biased blow fly pollinators. Phytochemistry, 153, 120–128. 10.1016/j.phytochem.2018.06.005. 29906658

[tpj14496-bib-0134] Wenke, K. , Kopka, J. , Schwachtje, J. , van Dongen, J.T. and Piechulla, B. (2019) Volatiles of rhizobacteria *Serratia* and *Stenotrophomonas* alter growth and metabolite composition of *Arabidopsis thaliana* . Plant Biol. 21, 109–119. 10.1111/plb.12878. 30030887

[tpj14496-bib-0135] Widhalm, J.R. , Jaini, R. , Morgan, J.A. and Dudareva, N. (2015) Rethinking how volatiles are released from plant cells. Trends Plant Sci. 20(9), 545–550. 10.1016/j.tplants.2015.06.009. 26189793

[tpj14496-bib-0136] Wong, D.C.J. , Pichersky, E. and Peakall, R. (2017) The biosynthesis of unusual floral volatiles and blends involved in orchid pollination by deception: current progress and future prospects. Front. Plant Sci. 8, 1955 10.3389/fpls.2017.01955. 29181016 PMC5693887

[tpj14496-bib-0137] Wong, D.C.J. , Amarasinghe, R. , Pichersky, E. and Peakall, R. (2018) Evidence for the involvement of fatty acid biosynthesis and degradation in the formation of insect sex pheromone‐mimicking chiloglottones in sexually deceptive *Chiloglottis* orchids. Front. Plant Sci. 9, 839 10.3389/fpls.2018.00839. 29971087 PMC6018206

[tpj14496-bib-0138] Wu, D. , Qi, T. , Li, W.‐X. , Tian, H. , Gao, H. , Wang, J. , Ge, J. , Yao, R. , Ren, C. and Wang, X.‐B. (2017) Viral effector protein manipulates host hormone signaling to attract insect vectors. Cell Res. 27(3), 402. 28059067 10.1038/cr.2017.2 PMC5339842

[tpj14496-bib-0139] Wu, Q. , Tao, X. , Ai, X. , Luo, Z. , Mao, L. , Ying, T. and Li, L. (2018) Contribution of abscisic acid to aromatic volatiles in cherry tomato (Solanum lycopersicum L.) fruit during postharvest ripening. Plant Physiol. Biochem. 130, 205–214. 10.1016/j.plaphy.2018.06.039. 29990773

[tpj14496-bib-0140] Xu, J. , van Herwijnen, Z.O. , Drager, D.B. , Sui, C. , Haring, M.A. and Schuurink, R.C. (2018) SlMYC1 regulates type VI glandular trichome formation and terpene biosynthesis in tomato glandular cells. Plant Cell, 30(12), 2988–3005. 10.1105/tpc.18.00571. 30518626 PMC6354261

[tpj14496-bib-0141] Yamamoto, Y. , Negi, J. , Wang, C. , Isogai, Y. , Schroeder, J.I. and Iba, K. (2016) The transmembrane region of guard cell SLAC1 channels perceives CO_2_ signals via an ABA‐independent pathway in Arabidopsis. Plant Cell, 28(2), 557–567. 10.1105/tpc.15.00583. 26764376 PMC4790869

[tpj14496-bib-0142] Yan, H. , Baudino, S. , Caissard, J.‐C. , Florence, N. , Zhang, H. , Tang, K. , Li, S. and Lu, S. (2018) Functional characterization of the eugenol synthase gene (RcEGS1) in rose. Plant Physiol. Biochem. 129, 21–26. 10.1016/j.plaphy.2018.05.015. 29787935

[tpj14496-bib-0143] Ye, M. , Glauser, G. , Lou, Y. , Erb, M. and Hu, L. (2018) Molecular dissection of early defense signaling underlying volatile‐mediated defense priming and herbivore resistance in rice. BioRxiv, 31, 687–698. 10.1101/378752 PMC6482627 30760558

[tpj14496-bib-0144] Yoshida, K. , Oyama‐Okubo, N. and Yamagishi, M. (2018) An R2R3‐MYB transcription factor ODORANT1 regulates fragrance biosynthesis in lilies (*Lilium* spp.). Mol. Breed. 38(12), 144 10.1007/s11032-018-0902-2.

[tpj14496-bib-0145] Yu, Z.‐X. , Wang, L.‐J. , Zhao, B. , Shan, C.‐M. , Zhang, Y.‐H. , Chen, D.‐F. and Chen, X.‐Y. (2015) Progressive regulation of sesquiterpene biosynthesis in Arabidopsis and Patchouli (*Pogostemon cablin*) by the miR156‐targeted SPL transcription factors. Mol. Plant, 8(1), 98–110. 25578275 10.1016/j.molp.2014.11.002

[tpj14496-bib-0146] Zebelo, S.A. , Matsui, K. , Ozawa, R. and Maffei, M.E. (2012) Plasma membrane potential depolarization and cytosolic calcium flux are early events involved in tomato (*Solanum lycopersicon*) plant‐to‐plant communication. Plant Sci. 196, 93–100. 10.1016/j.plantsci.2012.08.006. 23017903

[tpj14496-bib-0147] Zhang, H. , Xie, Y. , Liu, C. , Chen, S. , Hu, S. , Xie, Z. , Deng, X. and Xu, J. (2017) Comprehensive comparative analysis of volatile compounds in citrus fruits of different species. Food Chem. 230, 316–326. 28407917 10.1016/j.foodchem.2017.03.040

[tpj14496-bib-0148] Zhang, J. , Wang, N. , Miao, Y. , Hauser, F. , McCammon, J.A. , Rappel, W.‐J. and Schroeder, J.I. (2018a) Identification of SLAC1 anion channel residues required for CO_2_/bicarbonate sensing and regulation of stomatal movements. Proc. Natl Acad. Sci. USA, 115(44), 11129–11137. 10.1073/pnas.1807624115. 30301791 PMC6217375

[tpj14496-bib-0149] Zhang, J. , Zeng, L. , Chen, S. , Sun, H. and Ma, S. (2018b) Transcription profile analysis of *Lycopersicum esculentum* leaves, unravels volatile emissions and gene expression under salinity stress. Plant Physiol. Biochem. 126, 11–21. 10.1016/j.plaphy.2018.02.016. 29482070

[tpj14496-bib-0150] Zhang, T. , Sun, M. , Guo, Y. , Shi, X. , Yang, Y. , Chen, J. , Zheng, T. , Han, Y. , Bao, F. and Ahmad, S. (2018c) Overexpression of *LiDXS* and *LiDXR* from lily (*Lilium* ‘siberia’) enhances the terpenoid content in tobacco flowers. Front. Plant Sci. 9, 909 10.3389/fpls.2018.00909. 30038631 PMC6046550

[tpj14496-bib-0151] Zhang, P.J. , Wei, J.N. , Zhao, C. , Zhang, Y.F. , Li, C.Y. , Liu, S.S. , Dicke, M. , Yu, X.P. and Turlings, T.C.J. (2019) Airborne host‐plant manipulation by whiteflies via an inducible blend of plant volatiles. Proc. Natl. Acad. Sci. USA, 116(15), 7387–7396. 10.1073/pnas.1818599116. 30910967 PMC6462071

[tpj14496-bib-0152] Zhou, Y. , Peng, Q. , Zeng, L. , Tang, J. , Li, J. , Dong, F. and Yang, Z. (2018) Study of the biochemical formation pathway of aroma compound 1‐phenylethanol in tea (*Camellia sinensis* (L.) O. Kuntze) flowers and other plants. Food Chem. 258, 352–358. 10.1016/j.foodchem.2018.03.095. 29655745

[tpj14496-bib-0153] Zhou, Y. , Peng, Q. , Zhang, L. , Cheng, S. , Zeng, L. , Dong, F. and Yang, Z. (2019) Characterization of enzymes specifically producing chiral flavor compounds (R)‐ and (S)‐1‐phenylethanol from tea (*Camellia sinensis*) flowers. Food Chem. 280, 27–33. 10.1016/j.foodchem.2018.12.035. 30642496

